# Iodine-Substituted Dithiocarbamic Flavanones—A Structure–Activity Relationship Study of Their Antioxidant Properties

**DOI:** 10.3390/molecules30112280

**Published:** 2025-05-22

**Authors:** Mihail Lucian Birsa, Laura Gabriela Sarbu

**Affiliations:** Department of Chemistry, Alexandru Ioan Cuza University of Iasi, 11 Carol I Blvd., 700506 Iasi, Romania

**Keywords:** antioxidants, benzopyrans, dithiocarbamates, flavanones, radical enolates, benzylic radical

## Abstract

The antioxidant properties of novel diiodo-substituted 3-dithiocarbamic flavanones were investigated. The three frameworks that proved to be the most active ones in our previous studies were selected. By varying the nature of the substituent at the para position of flavanone ring **B**, a structure–activity relationship study on radical scavenging properties was performed. The influence of these substituents (F, Cl, Br and H) was investigated against DPPH and ABTS^+•^. The results indicate that the presence of the halogen substituents induces better antioxidant properties than ascorbic acid and BHT. The highest radical scavenging activity was found in the case of morpholine carbodithioates. Regarding the ABTS^+•^ assay, all investigated flavanones exhibited better antioxidant properties than BHT.

## 1. Introduction

Due to their various biological properties, flavonoids have long captured the interest of biologists and chemists [1,2]. Important milestones in discovering and developing the health benefits of these compounds were provided by several studies performed in the 1990s [3,4,5,6]. Since then, these studies have been supported by the results of in vitro and in vivo studies, including human trials [7,8,9,10,11,12]. The health effects of dietary flavonoids have been reviewed in recent years [13,14,15,16]. More than 13,000 flavonoids have been identified to date [17]. Their comprehensive study from a dietary origin point of view has provided a series of reports and databases that present flavonoid contents in foods, the dietary level of consumption and their biotransformation and bioavailability [18,19,20]. Flavonoids have been shown to display cardioprotective, neuroprotective, antiproliferative, antiviral and antimicrobial properties [21,22,23,24,25,26,27].

**Figure 1:**
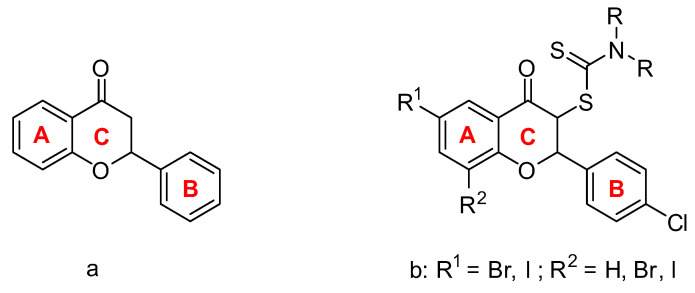
(**a**) The general structure of flavanones; (**b**) the structure of previously investigated flavanones.

From a chemical point of view, the term flavonoid refers to those molecules the structural C6–C3–C6 backbone (Figure 1) of which consists of two benzene rings (**A** and **B**) that are linked through three carbon atoms that form a benzopyran heterocyclic ring (**C**). This structural versatility enables diverse substitution patterns, giving rise to various flavonoid subclasses such as flavonols, flavones, flavanones, flavanols and anthocyanidins [28].

Reactive oxygen species (ROS) are unstable derivatives of molecular oxygen, which display high reactivity. ROS are divided into two main categories, namely radical ROS, such as superoxide (O_2_^•−^), peroxyl (HO•) or hydroxyl radicals (ROO•), and non-radical ROS, such as singlet oxygen (^1^O_2_) and hydrogen peroxide (H_2_O_2_). In humans, ROS are generated mainly by mitochondrial processes, as well as by various enzymes during normal cell metabolism. At low concentrations, ROS can act as mediators in a number of signaling processes; however, at higher concentrations, ROS indiscriminately damage biomacromolecules such as DNA, proteins, lipids and carbohydrates. In the human body, this kind of oxidative stress can be counteracted to some degree with the help of antioxidants. These in turn can be produced by the cells (endogenous) and include enzymes, non-enzymatic proteins, thiols, etc., or acquired from the diet (exogenous), such as vitamins, carotenoids, polyphenols, etc. The recognition of the antioxidant properties of flavonoids derives from generalized terms that “flavonoids act primarily as antioxidant molecules” [29,30,31]. The impact of flavonoids on the cell’s antioxidant capacity has been reported to take place through several mechanisms [32,33,34,35,36,37,38,39,40].

Recently, we described the synthesis and antimicrobial and cytotoxic properties of several novel synthetic flavonoids bearing a 1,3-dithiolium ring fused to the **C** ring of the flavan core [41,42,43,44,45,46,47]. In order to extend our previous investigations on the antioxidant properties of halogen-substituted 3-dithiocarbamic flavanones, we are reporting here the antioxidant behavior of novel synthetic dithiocarbamic flavanones bearing two iodine substituents on the **A** ring, used as precursors in the synthesis of the above-mentioned tricyclic synthetic flavonoids.

## 2. Results

### 2.1. 3-Dithiocarbamic Flavanones

**Scheme 1:**
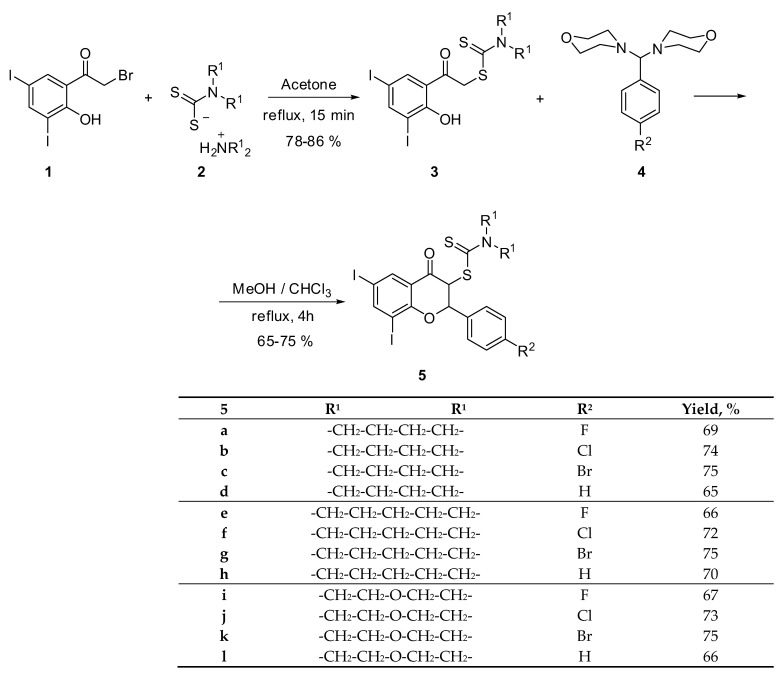
Synthesis of 3-dithiocarbamic flavanones **5a**–**l**.

The synthesis of 3-dithiocarbamic flavanones **5a**–**l** involves two steps, as described in Scheme 1. The first consists of the synthesis of the corresponding diiodo-substituted phenacyl dithiocarbamates **3**. This was accomplished by the reaction of 2-bromo-1-(3,5-diiodo-2-hydroxyphenyl)ethan-1-one **1** [48] with various salts of *N*,*N*-dialkyldithiocarbamic acid **2**, in good yields (78–86%), as previously reported by us [49]. The synthesis of the latter compounds was performed by the reaction of carbon disulfide with pyrrolidine, piperidine and morpholine, denoted as R^1^ in Scheme 1 [50]. In a second step, the treatment of diiodo-substituted phenacyl carbodithioates **3** with various para-substituted aminals **4** was performed according to an experimental procedure reported by us [51]. The target 3-dithiocarbamic flavanones **5a**–**l** were obtained in good yields (65–75%) (Scheme 1). The synthesis of flavanones **5b**, **5f** and **5j** has recently been reported by us [52].

The structure of these novel 3-dithiocarbamic flavanones was proved by analytical and spectral data. The data from the elemental analyses are presented in the Supplementary Material. The heterocyclocondensation to the benzopyran ring is confirmed by the spectral changes. Thus, the ^1^H NMR spectra show the disappearance of the phenolic proton signal, as well as of the methylene protons signal from ca. 4.8 ppm, and the appearance of two new doublets ranging from 6.2 ppm to 5.5 ppm, corresponding to the hydrogen atoms from the C-2 and C-3 positions of the benzopyran ring. The NMR pattern of the para-substituted aromatic ring originating from aminals **4** can also be observed. Furthermore, the ^13^C NMR spectra also support the closure of the benzopyran ring. The appearance of a new signal provided by the thiocarbonyl carbon atom around 186–190 ppm and the presence of the C-2 carbon atom, found around 81 ppm, and the C-3 carbon atom at ca. 58 ppm also confirm the structure of 3-dithiocarbamic flavanones **5**.

**Figure 2:**
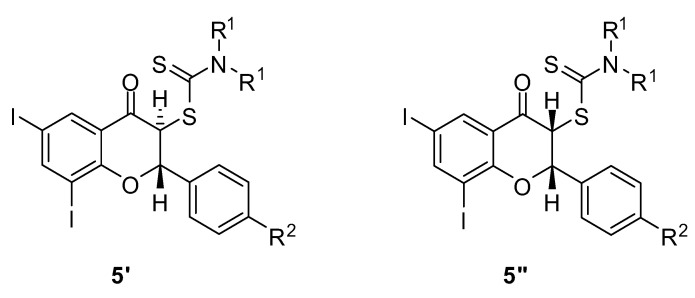
Diastereoisomers of flavanones **5**.

From a stereochemical perspective, these flavanones exhibit two diastereoisomers arising from the relative orientation of H-2 and H-3 hydrogen atoms. It should be mentioned that the two diastereisomers are obtained as an inseparable mixture. These two atoms can be positioned either on opposite sides or the same side of the benzopyran core (Figure 2). The relative orientations of these two substituents are reflected on the magnitude of their coupling constants. As expected, the most stable isomer should be that with an *anti* orientation of the two hydrogen atoms (**5′**) as opposed to the *syn* diastereomer **5**″. However, it was found that in the case of flavanone **5g** the *syn* isomer is the major one in the isolated mixture of isomers. The diastereoisomeric ratio and coupling constants of the flavanones **5** are presented in Table 1.

### 2.2. In Vitro Antioxidant Activity

Investigations of the antioxidant properties of the above-synthesized compounds was performed by using DPPH and ABTS^+•^ scavenging assays. BHT and ascorbic acid, two common antioxidants, were used as standards for the antioxidant potential comparison [53,54,55]. The experimental procedures are described in Section 4.2. The obtained results are presented in Table 2.

## 3. Discussion

Our previous studies on the antioxidant properties of 3-dithiocarbamic flavanones focused on the structure–activity relationship in terms of investigating the influence of different substituents at the **A** ring and different dithiocarbamic moieties, while keeping the same substituent (chlorine) at the para position of the **B** ring (Figure 1) [56]. These studies revealed that, among the investigated dithiocarbamic flavanones, namely 6-bromo, 6,8-dibromo and 6,8-diiodo, those that have two substituents on the **A** ring, possess the highest antioxidant activity. With regard to the nature of the secondary amine moiety, the pyrrolidinyl, piperidinyl and morpholinyl derivatives were found to be the most active substituted flavanones.

Based on these findings, we decided to further investigate the radical scavenging activity of three new sets of dithiocarbamic flavanones, where the 6,8-diiodo pattern on the **A** ring is combined with the above-mentioned dithiocarbamic secondary amine moieties. By varying the nature of the substituent at the para position of the **B** ring, a structure–activity relationship study was realized (Scheme 1). According to the data presented in Table 2, the radical scavenging activity of flavanones **5a–l** against DPPH indicated better antioxidant properties when compared to BHT, for all of the tested compounds which showed smaller IC_50_ values than BHT. The morpholine carbodithioates **5i–l** were found to be the most active compounds, with better antioxidant properties than both ascorbic acid and BHT.

Regarding the ABTS^+•^ radical scavenging assay, all tested flavanones performed better than both reference materials. Although all 12 investigated flavanones showed very good antioxidant properties, the activity of the compounds **5f**, **5h**, **5g** and **5k** is worth underlining. Both flavanones **5g** and **5k** have a bromine substituent at the para position of ring **B**.

Regardless, the lack of a direct connection between the nature of the para substituent at the aforementioned ring and the antioxidant properties of investigated flavanones, the data presented in Table 2 indicate that bromine and unsubstituted substrates at ring B are the most active ones.

**Scheme 2:**
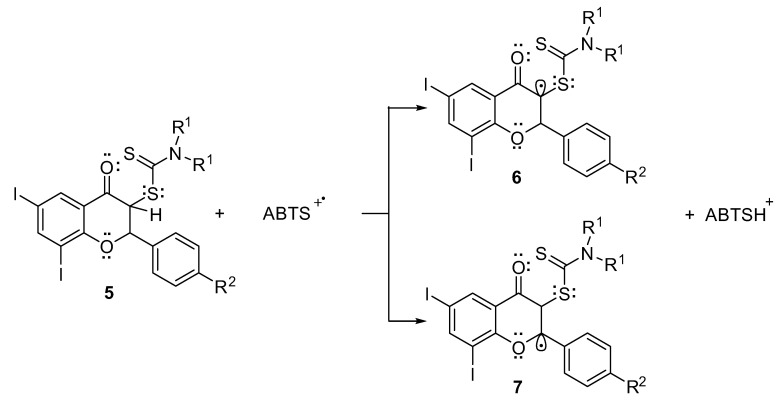
A tentative radical scavenging mechanism of flavanones **5**.

Although the flavanones of type **5** were obtained as an inseparable mixture of diastereomers, the antioxidant capacity does not depend on the stereochemistry of the *syn* and *anti* isomers. In our previous reports about the antioxidant properties of 3-dithiocarbamic flavanones, we provided some insights regarding their mechanism of action [57]. We presented evidence regarding the existence of an enolate radical at the C-3 carbon atom of the benzopyran ring (**6**, Scheme 2) and also the formation of a benzylic free radical at the C-2 position (**7**, Scheme 2). As the geometry of the free radicals is a pyramidal one, which can suffer inversion through a planar structure, the stereochemistry of the *syn* and *anti* isomers is missing. Consequently, the stereochemistry at the C-2 and C-3 carbon atoms from the flavanones **5** does not make any difference in terms of the antioxidant properties. Furthermore, the presence of the dithiocarbamic moiety at the C-3 position induces a supplementary stabilization of the enolate radical. It seems that, in the case of the described compounds, the presence of an iodine substituent at the C-8 position of the benzopyran ring decreases the stability of a benzyl-type radical at the C-2 position and thus the electronic effects of the para substituents from ring **B** are diminished. However, the existence of both free radicals at C-2 and C-3 on the same intermediate cannot be ruled out.

## 4. Materials and Methods

### 4.1. Chemistry

Melting points were obtained on a *KSPI* melting point meter (A. KRÜSS Optronic, Hamburg, Germany) and are uncorrected. IR spectra were recorded on a Bruker Tensor 27 instrument (Bruker Optik GmbH, Ettlingen, Germany). UV–vis measurements were carried out using a Varian BioChem 100 spectrophotometer (Varian, Sydney, Australia) equipped with an 8 × 6 multicell thermostatted water block. NMR spectra were recorded on a Bruker 500 MHz spectrometer (Bruker BioSpin, Rheinstetten, Germany). Chemical shifts are reported for the major isomer in ppm downfield from TMS. Mass spectra were recorded on a Thermo Scientific ISQ LT instrument (Thermo Fisher Scientific Inc., Waltham, MA, USA). All reagents were commercially available (Carl Roth) and used without further purification.

#### 4.1.1. General Procedure for 1-(2-Hydroxy-3,5-diiodophenyl)-1-oxaethan-2-yl-dithiocarbamates **3**

To a solution of 2-bromo-1-(2-hydroxy-3,5-diiodophenyl)ethan-1-one (**1**, 5 mmol) in acetone (25 mL), a solution of the corresponding ammonium dithiocarbamate **2** in acetone/water (10 mL/10 mL) was added. The resulting mixture was refluxed for 10 min, cooled down to room temperature and poured into water (200 mL) under vigorous stirring. The precipitate thus formed was vacuum-filtered and recrystallized from ethanol, yielding dithiocarbamates **3** as yellow crystals. The analytical and spectral data are identical to those previously reported by us [58].

#### 4.1.2. General Procedure for 6,8-Diiodo-2-(4-fluorophenyl)-4-oxochroman-3-yl-pyrrolidine-1-carbodithioate (**5a**)

To a solution of 1-(3,5-diiodo-2-hydroxyphenyl)-1-oxoethan-2-yl-pyrrolidine-1-carbodithioate (**3a**) (0.53 g, 1 mmol) in a mixture of MeOH/CHCl_3_ (20 mL, 1:1 *v*/*v*), aminal **4** (R^2^ = F, 0.28 g, 1 mmol) was added, and the reaction mixture was heated under reflux for 4 h. After cooling, the solid material was filtered off and purified by recrystallization from ethanol to give dithiocarbamate **5a** (0.44 g, 69%) as pale yellow crystals. M.p. 170–171 °C. IR (ATR, cm^−1^) 2988, 1488, 1248, 1109, 901, 605. ^1^H NMR (CDCl_3_, selected data for the major *anti* isomer) δ 8.27 (d, *J* = 2.1 Hz, 1H), 8.15 (d, *J* = 2.1 Hz, 1H), 7.51 (m, 2H), 7.16 (m, 2H), 5.99 (d, *J* = 10 Hz, 1H), 5.78 (d, *J* = 10 Hz, 1H), 3.91 (m, 2H), 3.65 (m, 2H), 2.08 (m, 2H), 1.99 (m, 2H). ^13^C NMR (CDCl_3_, selected data for the major *anti* isomer) δ 188.0, 185.9, 163.0, 158.7, 152.7, 136.4, 129.4, 115.6, 87.5, 85.0, 82.4, 57.7, 55.9, 50.9, 26.1, 24.3. MS (EI) *m*/*z*: 638.91 (M^+^, 6%) for C_20_H_16_FI_2_NO_2_S_2_.

#### 4.1.3. 6,8-Diiodo-2-(4-bromophenyl)-4-oxochroman-3-yl-pyrrolidine-1-carbodithioate (**5c**)

Yield 0.52 g, 75%. M.p. 184–183 °C. IR (ATR, cm^−1^) 2857, 1588, 1451, 1231, 1112, 1005, 839, 651. ^1^H NMR (DMSO-*d*_6_, selected data for the major *anti* isomer) δ 8.40 (d, *J* = 2.0 Hz, 1H), 8.01 (d, *J* = 2.0 Hz, 1H), 7.61 (d, *J* = 8.4 Hz, 2H), 7.51 (d, *J* = 8.4 Hz, 2H), 6.21 (d, *J* = 10.5 Hz, 1H), 5.82 (d, *J* = 10.5 Hz, 1H), 3.70 (m, 4H), 1.90 (m, 4H). ^13^C NMR (DMSO-*d*_6_, selected data for the major *anti* isomer) δ 186.4, 186.1, 158.9, 152.3, 135.9, 135.6, 88.8, 86.8, 82.0, 56.7, 56.4, 51.5, 25.9, 24.1. MS (EI) *m*/*z*: 745.7 (M^+^, 3%) for C_20_H_16_^79^BrI_2_NO_2_S_2_.

#### 4.1.4. 6,8-Diiodo-2-phenyl-4-oxochroman-3-yl-pyrrolidine-1-carbodithioate (**5d**)

Yield (0.41 g, 65%). M.p. 138–139 °C. IR (ATR, cm^−1^) 2871, 1696, 1468, 1225, 989, 803, 645, 542. ^1^H NMR (CDCl_3_, selected data for the major *anti* isomer) δ 8.26 (d, *J* = 1.8 Hz, 1H), 8.13 (d, *J* = 1.8 Hz, 1H), 7.52 (m, 2H), 7.38 (m, 3H), 6.04 (d, *J* = 6.8 Hz, 1H), 5.75 (d, *J* = 6.8 Hz, 1H), 3.91 (m, 2H), 3.63 (m, 2H), 2.07 (m, 2H), 1.99 (m, 2H). ^13^C NMR (CDCl_3_, selected data for the major *anti* isomer) δ 188.1, 186.0, 158.8, 152.7, 136.4, 135.7, 128.7, 127.2, 87.5, 84.9, 82.9, 57.5, 57.2, 50.9, 26.1, 24.3. MS (EI) *m*/*z*: 620.81 (M^+^, 5%) for C_20_H_17_I_2_NO_2_S_2_.

#### 4.1.5. 6,8-Diiodo-2-(4-fluorophenyl)-4-oxochroman-3-yl-piperidine-1-carbodithioate (**5e**)

Yield (0.48 g, 66%). M.p. 170–171 °C. IR (ATR, cm^−1^) 2847, 1656, 1435, 1225, 715, 658, 651. ^1^H NMR (DMSO-*d*_6_, selected data for the major *anti* isomer) δ 8.40 (d, *J* = 2.8 Hz, 1H), 8.01 (d, *J* = 2.8 Hz, 1H), 7.55 (m, 2H), 7.28 (m, 2H), 6.22 (d, *J* = 10.6 Hz, 1H), 5.88 (d, *J* = 10.6 Hz, 1H), 4.11 (m, 2H), 3.78 (m, 2H), 1.60 (m, 6H). ^13^C NMR (DMSO-*d*_6_, selected data for the major *anti* isomer) δ 190.4, 186.3, 163.0, 159.0, 152.1, 135.6, 129.5, 115.6, 89.9, 86.7, 82.2, 58.8, 57.2, 54.1, 26.2, 25.6, 23.8. MS (EI) *m*/*z*: 652.91 (M^+^, 9%) for C_21_H_18_FI_2_NO_2_S_2_.

#### 4.1.6. 6,8-Diiodo-2-(4-bromophenyl)-4-oxochroman-3-yl-piperidine-1-carbodithioate (**5g**)

Yield (0.52 g, 75%). M.p. 198–199 °C. IR (ATR, cm^−1^) 2826, 1678, 1499, 1458, 1205, 839, 705, 615. ^1^H NMR (CDCl_3_, selected data for the major *syn* isomer) δ 8.26 (m, 1H), 8.22 (m, 1H), 7.52 (d, *J* = 8.1 Hz, 2H), 7.41 (d, *J* = 8.1 Hz, 2H), 6.32 (d, *J* = 3.5 Hz, 1H), 5.98 (d, *J* = 3.5 Hz, 1H), 4.26 (m, 2H), 3.83 (m, 2H), 1.69 (m, 6H). ^13^C NMR (CDCl_3_, selected data for the major *syn* isomer) δ 190.4, 186.3, 158.3, 152.6, 133.9, 131.7, 128.7, 123.1, 122.9, 122.1, 87.5, 85.3, 81.0, 58.0, 57.1, 26.0, 25.3, 24.1. MS (EI) *m*/*z*: 712.81 (M^+^, 5%) for C_21_H_18_^79^BrI_2_NO_2_S_2_.

#### 4.1.7. 6,8-Diiodo-2-phenyl-4-oxochroman-3-yl-piperidine-1-carbodithioate (**5h**)

Yield (0.48 g, 70%). M.p. 158–159 °C. IR (ATR, cm^−1^) 2814, 1684, 1444, 1251, 1231, 799, 651. ^1^H NMR (DMSO-*d*_6_, selected data for the major *anti* isomer) δ 8.41 (d, *J* = 2.2 Hz, 1H), 8.03 (d, *J* = 2.2 Hz, 1H), 7.49 (m, 2H), 7.36 (m, 3H), 6.22 (d, *J* = 9.7 Hz, 1H), 5.86 (d, *J* = 9.7 Hz, 1H), 4.11 (m, 2H), 3.77 (m, 2H), 1.59 (m, 6H). ^13^C NMR (DMSO-*d*_6_, selected data for the major *anti* isomer) δ 190.4, 186.3, 159.0, 152.2, 136.6, 135.6, 129.5, 128.9, 122.0, 89.9, 86.7, 82.8, 58.3, 57.2, 26.5, 25.6, 23.8. MS (EI) *m*/*z*: 634.90 (M^+^, 2%) for C_21_H_19_I_2_NO_2_S_2_.

#### 4.1.8. 6,8-Diiodo-2-(4-fluorophenyl)-4-oxochroman-3-yl-morpholine-4-carbodithioate (**5i**)

Yield (0.46 g, 67%). M.p. 178–179 °C. IR (ATR, cm^−1^) 2869, 1712, 1602, 1444, 1258, 805, 512. ^1^H NMR (DMSO-*d*_6_, selected data for the major *anti* isomer) δ 8.40 (d, *J* = 2.2 Hz, 1H), 8.02 (d, *J* = 2.2 Hz, 1H), 7.59 (m, 2H), 7.26 (m, 2H), 6.24 (d, *J* = 10.3 Hz, 1H), 5.88 (d, *J* = 10.3 Hz, 1H), 4.14 (m, 4H), 3.58 (m, 4H). ^13^C NMR (DMSO-*d*_6_, selected data for the major *anti* isomer) δ 192.2, 186.1, 162.0, 159.0, 152.2, 135.6, 130.5, 115.7, 89.8, 86.8, 82.1, 65.9, 56.9, 51.5. MS (EI) *m*/*z*: 654.88 (M^+^, 2%) for C_20_H_16_FI_2_NO_3_S_2_.

#### 4.1.9. 6,8-Diiodo-2-(4-bromophenyl)-4-oxochroman-3-yl-morpholine-4-carbodithioate (**5k**)

(0.52 g, 75%). M.p. 201–202 °C. IR (ATR, cm^−1^) 2925, 1715, 1599, 1454, 1247, 1214, 812, 625, 531. ^1^H NMR (DMSO-*d*_6_, selected data for the major *anti* isomer) δ 8.42 (d, *J* = 2.2 Hz, 1H), 8.02 (d, *J* = 2.2 Hz, 1H), 7.64 (m, 2H), 7.50 (m, 2H), 6.23 (d, *J* = 10.3 Hz, 1H), 5.88 (d, *J* = 10.3 Hz, 1H), 4.31 (m, 4H), 3.59 (m, 4H). ^13^C NMR (DMSO-*d*_6_, selected data for the major *anti* isomer) δ 192.1, 186.0, 158.9, 152.2, 135.9, 135.6, 131.8, 130.5, 122.9, 122.2, 89.8, 86.8, 82.1, 65.9, 58.3, 56.7. MS (EI) *m*/*z*: 714.79 (M^+^, 3%) for C_20_H_16_^79^BrINO_3_S_2_.

#### 4.1.10. 6,8-Diiodo-2-phenyl-4-oxochroman-3-yl-morpholine-4-carbodithioate (**5l**)

Yield (0.43 g, 66%). M.p. 157–158 °C. IR (ATR, cm^−1^) 2921, 1725, 1544, 1444, 1250, 1218, 809, 661, 534. ^1^H NMR (DMSO-*d*_6_, selected data for the major *anti* isomer) δ 8.42 (d, *J* = 2.1 Hz, 1H), 8.04 (d, *J* = 2.1 Hz, 1H), 7.51 (m, 2H), 7.42 (m, 3H), 6.24 (d, *J* = 9.5 Hz, 1H), 5.87 (d, *J* = 9.5 Hz, 1H), 4.32 (m, 4H), 3.61 (m, 4H). ^13^C NMR (DMSO-*d*_6_, selected data for the major *anti* isomer) δ 192.3, 186.1, 159.0, 152.3, 136.5, 135.5, 129.5, 128.5, 128.0, 121.9, 89.9, 86.7, 82.7, 65.9, 58.0, 56.8. MS (EI) *m*/*z*: 636.88 (M^+^, 1%) for C_20_H_17_I_2_NO_3_S_2_.

### 4.2. In Vitro Antioxidant Activities

#### 4.2.1. 2,2-Diphenyl-1-picrylhydrazyl (DPPH) Radical Assay

The DPPH method was performed according to the reported literature procedure [59,60]. Using a standard 4 mL quartz UV cell, flavanones **5a**–**l**, at different concentrations (1.4–2.1 mM in ethanol), were added to a solution of DPPH (0.1 mM in ethanol, 2.75 mL). The flavanones were added in 70 µL batches until absorbance dropped below half of the initial value. The reaction for scavenging DPPH radicals was performed in the dark at room temperature and the absorbance was measured at 517 nm after 15 min of incubation at 37 °C. The IC_50_ (nM) was determined as the flavanones’ **5a**–**l** concentration of 50% of the DPPH scavenging capacity (Table 1).

#### 4.2.2. 2,2′-Azino-bis(3-Ethylbenzothiazoline-6-sulfonic Acid) (ABTS) Assay

The ABTS assay was performed according to a slightly modified literature-reported protocol [61,62]. ABTS radical cation (ABTS^+•^) was prepared by reacting an ABTS solution (7 mM) in phosphate buffer (PBS, 0.1 M, pH 7.4) with a solution of potassium persulfate (2.45 mM) in water. The mixture was left to stand at room temperature for 16 h before use. After that, the mixture was diluted with PBS to reach an absorbance value of 0.70 ± 0.5 at 734 nm. Using a standard 4 mL quartz UV cell, flavanones **5a**–**l**, at different concentrations (1.5–2.2 mM in ethanol), were added to a solution of ABTS^+•^ (2.75 mL). The flavanones were added in 15 µL batches until absorbance dropped below half of the initial value. The reaction for scavenging ABTS^+•^ radicals was performed in the dark at room temperature and the absorbance was measured at 734 nm after 15 min of incubation at 37 °C. The IC_50_ (nM) was determined as the flavanones’ **5a**–**l** concentration of 50% of the ABTS^+•^ scavenging capacity (Table 1).

## 5. Conclusions

The antioxidant properties of novel iodine-substituted 3-dithiocarbamic flavanone frameworks were investigated. By changing the nature of the substituent at the para position of flavanone ring **B** (F, Cl, Br and H), a structure–activity relationship study of the radical scavenging activity was performed against DPPH and ABTS^+•^. The results indicate that the presence of the halogen substituents induces better antioxidant properties than ascorbic acid and BHT. For the DPPH assay, the iodine flavanones **5i–l** with a morpholine carbodithioate moiety were identified as the most active compounds. All investigated flavanones exhibited better antioxidant properties than BHT. On the other hand, the ABTS^+•^ assay showed that all of the tested compounds have better antioxidant activity than ascorbic acid and BHT. In the latter case, flavanones **5d**, **5g** and **5k** are, respectively, 2.5-, 3.5- and 1.5-fold more active than BHT.

## Figures and Tables

**Table 1:** Coupling constants and diastereoisomer ratio of flavanones **5**.

Flavanones 5	a	b	c	d	E	f	g	h	i	j	k	l
^3^*J*_H2–H3_*syn* (Hz)	3.8	3.5	2.5	4.0	2.6	2.9	3.5	1.6	1.3	2.5	2.9	2.2
*δ* H2, H3 (ppm)	5.99, 6.25	5.89, 6.41	5.89, 6.39	6.04, 6.29	5.98, 6.32	6.01, 6.33	5.98, 6.32	6.00, 6.38	5.95, 6.40	5.95, 6.42	5.95, 6.40	5.99, 6.41
^3^*J*_H2–H3_*anti* (Hz)	10.1	9.8	10.5	6.8	10.6	8.9	8.9	9.7	10.3	9.1	10.3	9.5
*δ* H2, H3 (ppm)	5.78, 5.99	5.82, 6.25	5.82, 6.21	5.52, 6.06	5.88, 6.23	5.78, 5.99	5.77, 5.98	5.86, 6.22	5.88, 6.24	5.88, 6.23	5.88, 6.23	5.87, 6.24
*syn: anti* ratio	28:72	35:65	48:52	20:80	42:58	40:60	67:33	43:57	47:53	33:67	46:54	45:55

**Table 2:** Radical scavenging activities of compounds **5a**–**l** (IC_50_ values in nM) *.

R^1^	R^1^	Compound	DPPH	ABTS^+•^
-CH_2_-CH_2_-CH_2_-CH_2_-		**5a**	277.00 ± 0.2	91.35 ± 0.5
		**5b**	273.84 ± 0.3	98.39 ± 0.4
		**5c**	264.37 ± 0.5	79.85 ± 0.3
		**5d**	291.84 ± 0.4	58.69 ± 0.4
-CH_2_-CH_2_-CH_2_-CH_2_-CH_2_-		**5e**	233.86 ± 0.4	123.51 ± 0.4
		**5f**	268.22 ± 0.2	71.80 ± 0.3
		**5g**	219.35 ± 0.3	44.85 ± 0.2
		**5h**	248.69 ± 0.3	87.53 ± 0.4
-CH_2_-CH_2_-O-CH_2_-CH_2_-		**5i**	173.54 ± 0.1	121.67 ± 0.3
		**5j**	158.27 ± 0.3	119.21 ± 0.3
		**5k**	174.55 ± 0.2	89.36 ± 0.2
		**5l**	145.87 ± 0.4	94.83 ± 0.4
Ascorbic acid			184.94 ± 0.2	147.47 ± 0.2
BHT			297.76 ± 0.2	148.29 ± 0.3

* Mean values from triplicate tests. Data are shown as the mean ± standard deviation.

## Data Availability

Data are contained within the article.

## Supplementary Materials

The following supporting information can be downloaded at: https://www.mdpi.com/article/10.3390/molecules30112280/s1. Elemental analysis and copies of ^13^C-NMR spectra.
