# Supplementary material for: Iodine-Substituted Dithiocarbamic Flavanones—A Structure–Activity Relationship Study of Their Antioxidant Properties
Source: Molecules. 2025 May 22;30(11):2280. doi: 10.3390/molecules30112280 (PMC12155936; doi:10.3390/molecules30112280)
Supplement: Supplementary file 1 [file molecules-30-02280-s001.zip › molecules-3606856-supplementary.pdf]

Iodine substituted dithiocarbamic flavanones – A structure-activity relationship study on the antioxidant properties

Mihail Lucian Birsa and Laura Gabriela Sarbu

Supplementary Material

1. Elemental analysis **S2**

2. Copies of ^{13}C NMR spectra **S3-S11**

3. Copies of ^{1}H NMR spectra **S12-S20**

1. Elemental analysis

Elemental analyses (C, H) were conducted using a CE440 Elemental Analyser; the results were found to be in good agreement (±0.3%) with the calculated values.

Table S1. Elemental analysis data for the newly synthesized flavanones **5a-l**.

Compound	% C		% H	
	calcd.	found	calcd.	found
**5a**	37.58	37.85	2.52	2.69
**5c**	34.31	34.58	2.30	2.52
**5d**	38.66	38.94	2.76	2.91
**5e**	38.61	38.91	2.78	3.02
**5g**	35.31	35.54	2.54	2.41
**5h**	39.70	39.88	3.01	2.84
**5i**	36.66	36.84	2.46	2.59
**5k**	33.54	33.79	2.25	2.41
**5l**	37.69	37.88	2.69	2.85

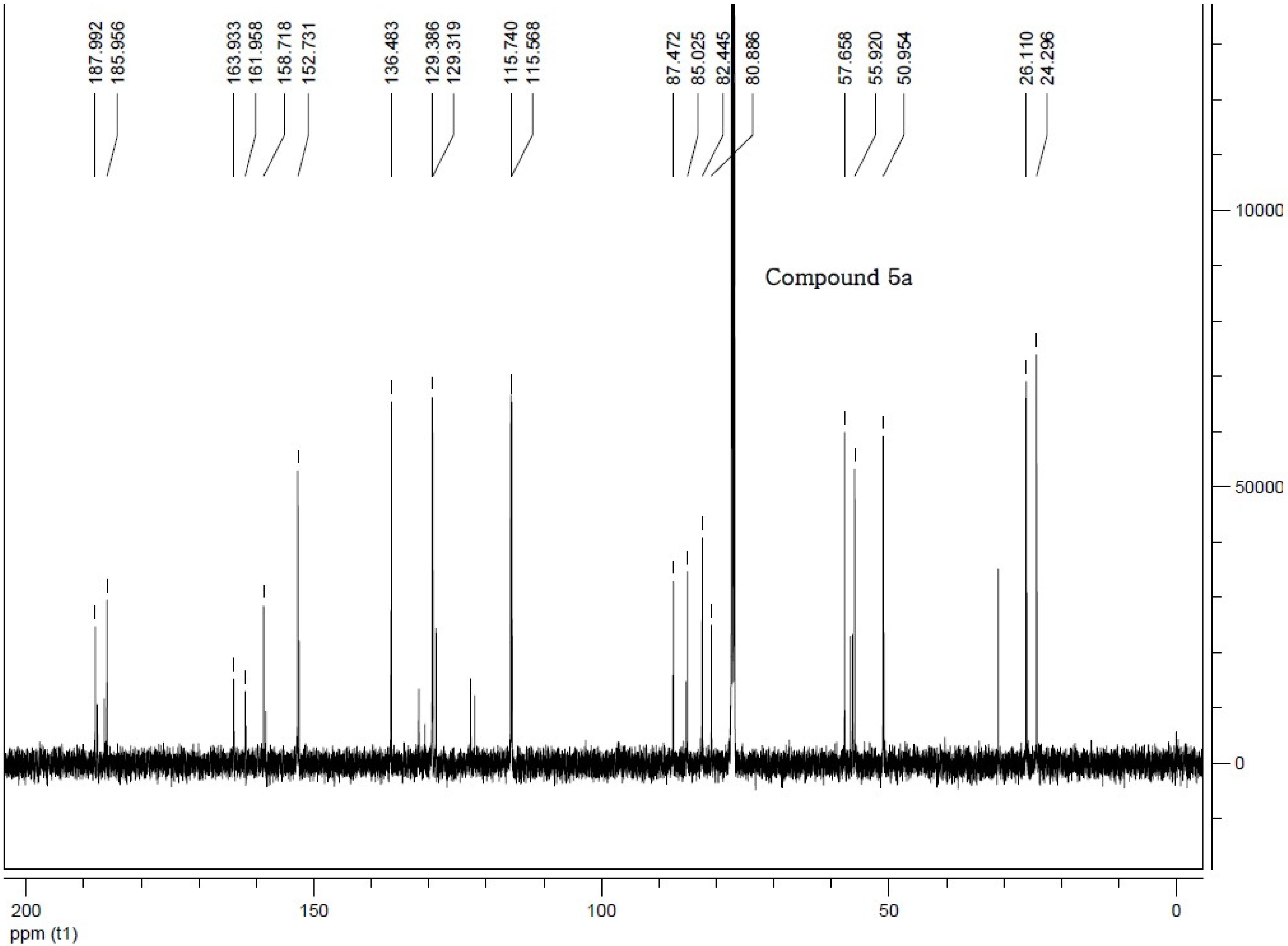
187.992
185.956
163.933
161.958
158.718
152.731
136.483
129.386
129.319
115.740
115.568
87.472
85.025
82.445
80.886
57.658
55.920
50.954
26.110
24.296
Compound 5a
10000
50000
0
200
150
100
50
0
ppm (t1)

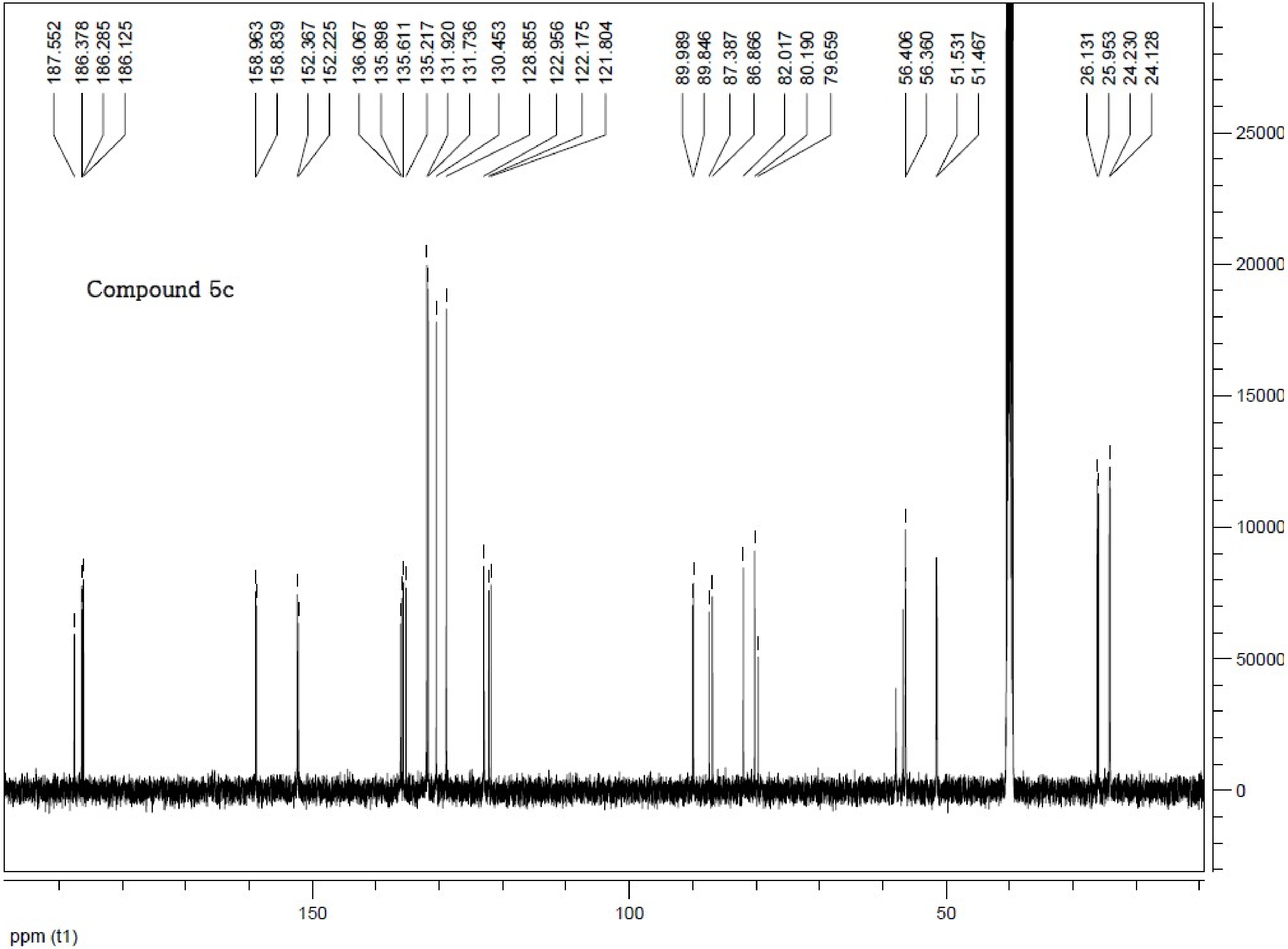
187.552
186.378
186.285
186.125
158.963
158.839
152.367
152.225
136.067
135.898
135.611
135.217
131.920
131.736
130.453
128.855
122.956
122.175
121.804
89.989
89.846
87.387
86.866
82.017
80.190
79.659
56.406
56.360
51.531
51.467
26.131
25.953
24.230
24.128
Compound 5c
25000
20000
15000
10000
50000
0
150
100
50
ppm (t1)

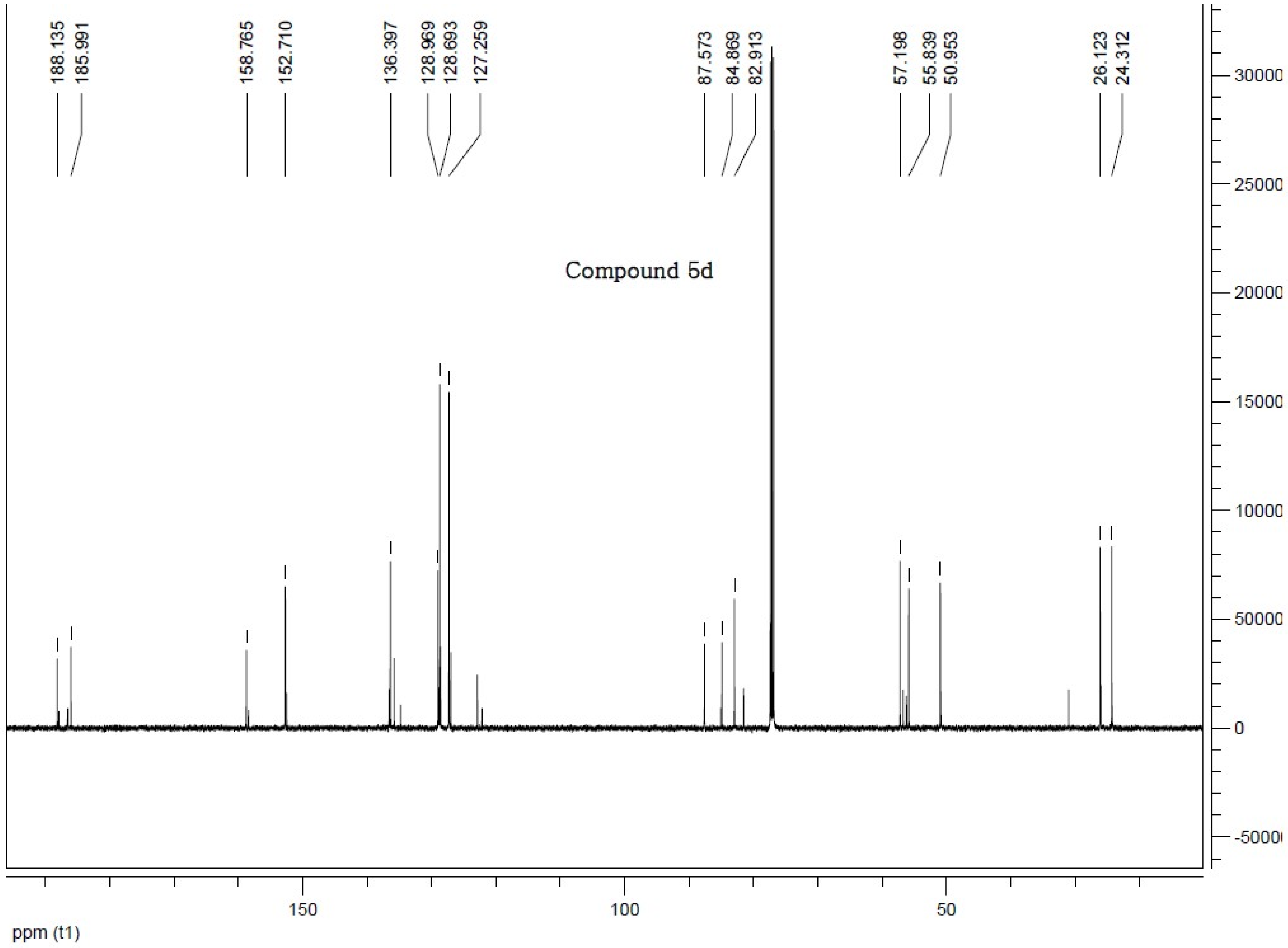
188.135
185.991
158.765
152.710
136.397
128.969
128.693
127.259
87.573
84.869
82.913
57.198
55.839
50.953
26.123
24.312
Compound 5d
30000
25000
20000
15000
10000
50000
0
-5000
150
100
50
ppm (t1)

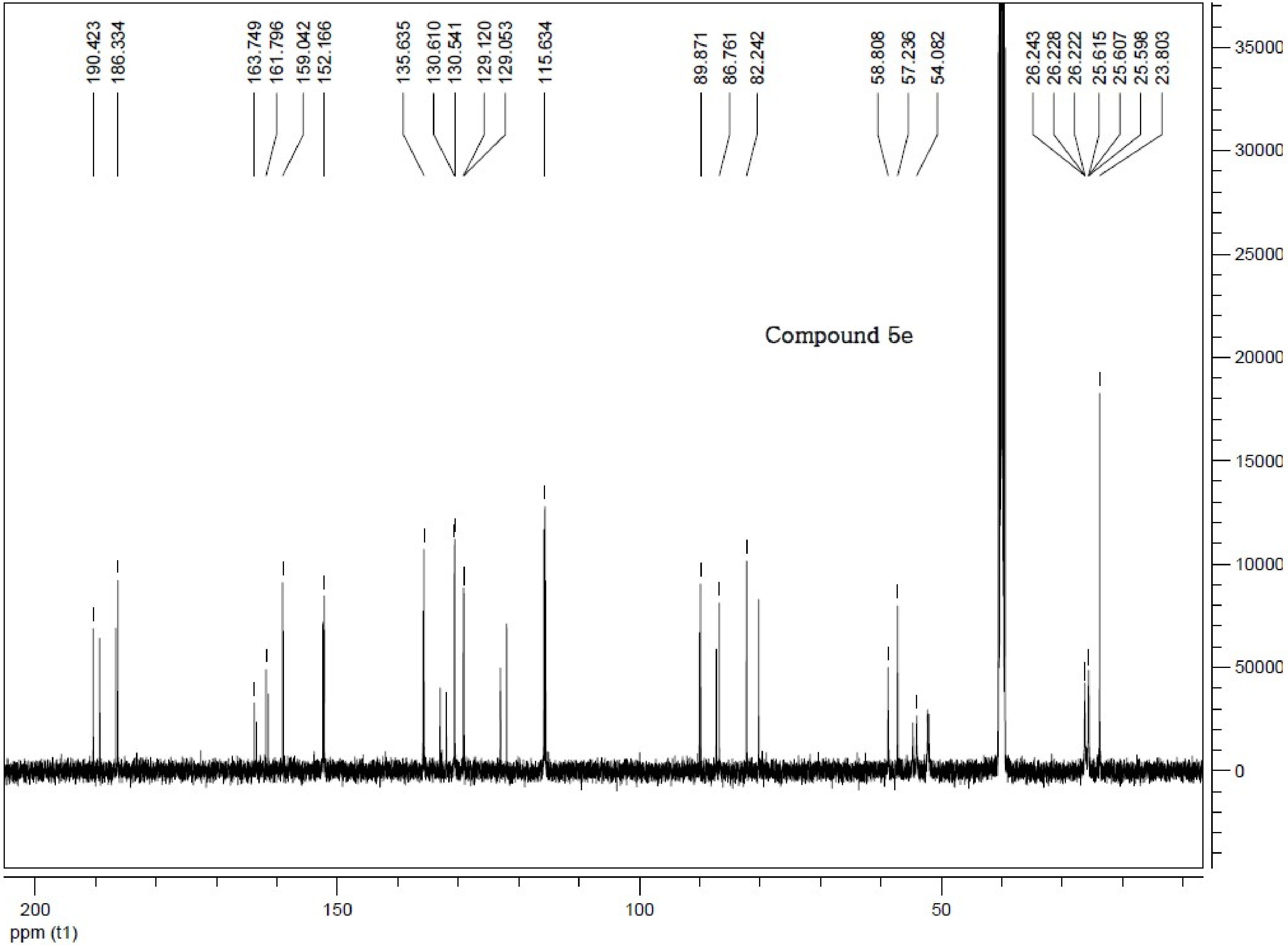

190.423
186.334
163.749
161.796
159.042
152.166
135.635
130.610
130.541
129.120
129.053
115.634
89.871
86.761
82.242
58.808
57.236
54.082
26.243
26.228
26.222
25.615
25.607
25.598
23.803
Compound 5e
35000
30000
25000
20000
15000
10000
50000
0
200
150
100
50
ppm (t1)

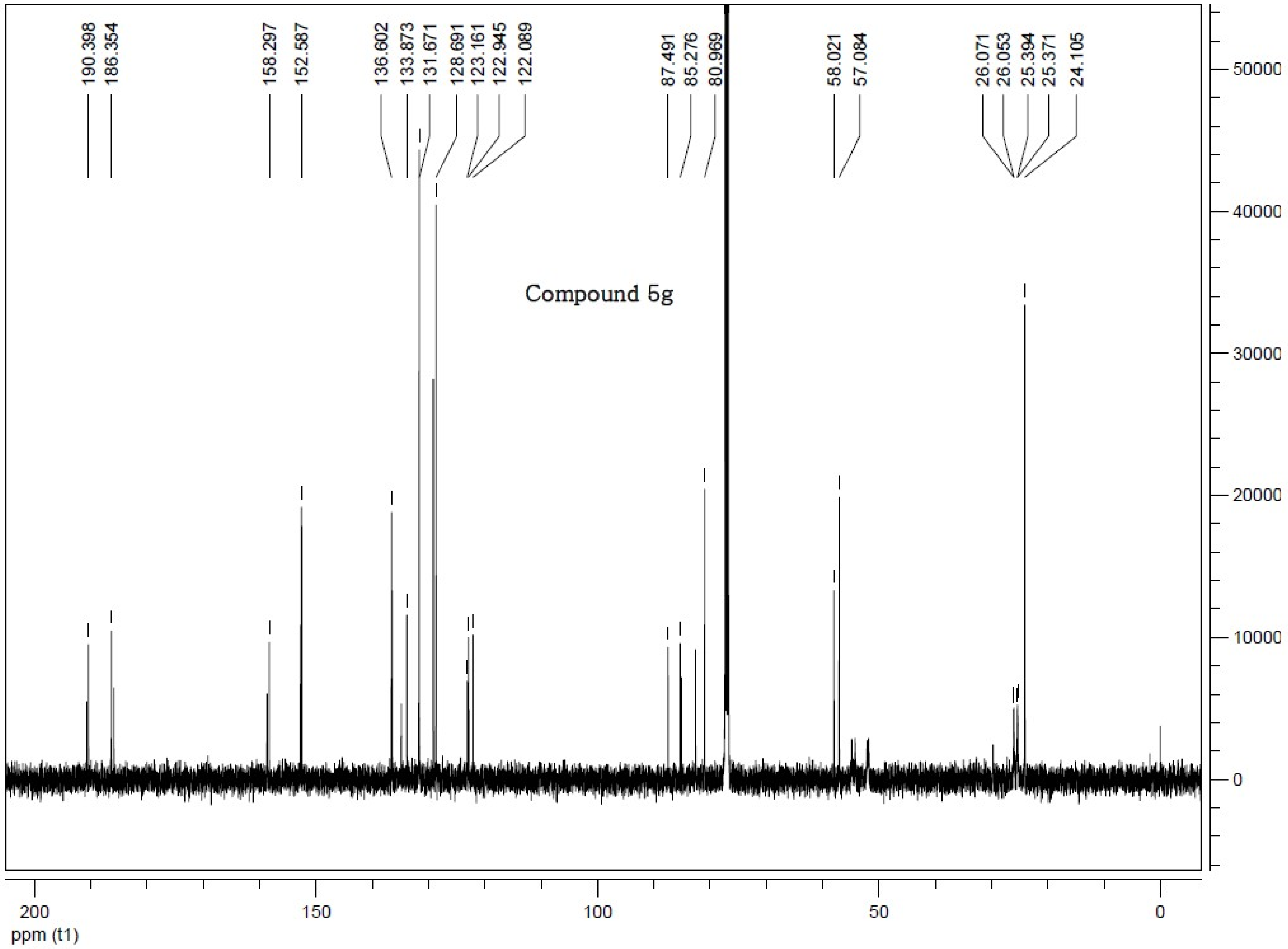
190.398
186.354
158.297
152.587
136.602
133.873
131.671
128.691
123.161
122.945
122.089
87.491
85.276
80.969
58.021
57.084
26.071
26.053
25.394
25.371
24.105
Compound 5g
50000
40000
30000
20000
10000
0
200
150
100
50
0
ppm (t1)

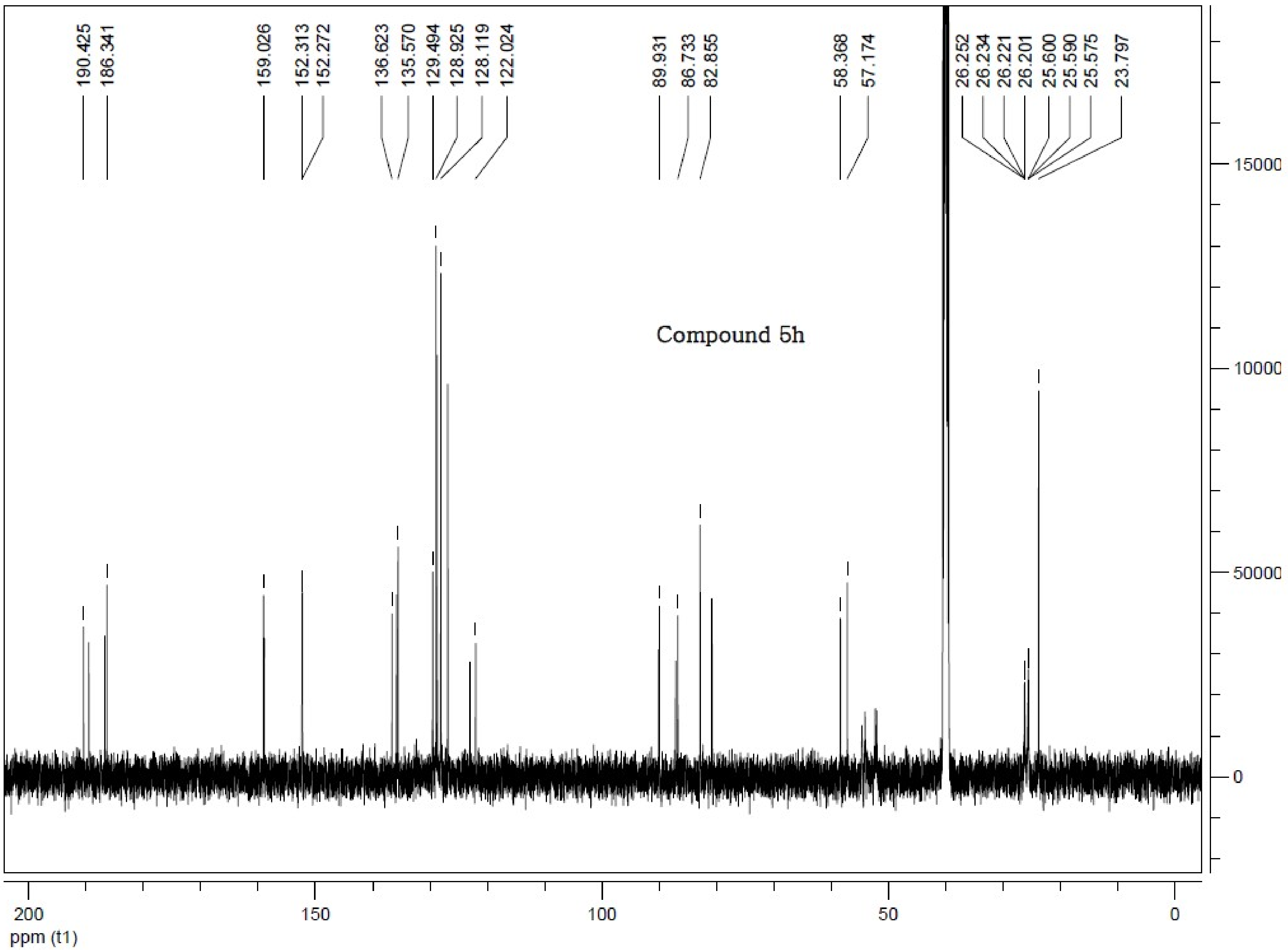
190.425
186.341
159.026
152.313
152.272
136.623
135.570
129.494
128.925
128.119
122.024
89.931
86.733
82.855
58.368
57.174
26.252
26.234
26.221
26.201
25.600
25.590
25.575
23.797
Compound 5h
15000
10000
50000
0
200
150
100
50
0
ppm (t1)

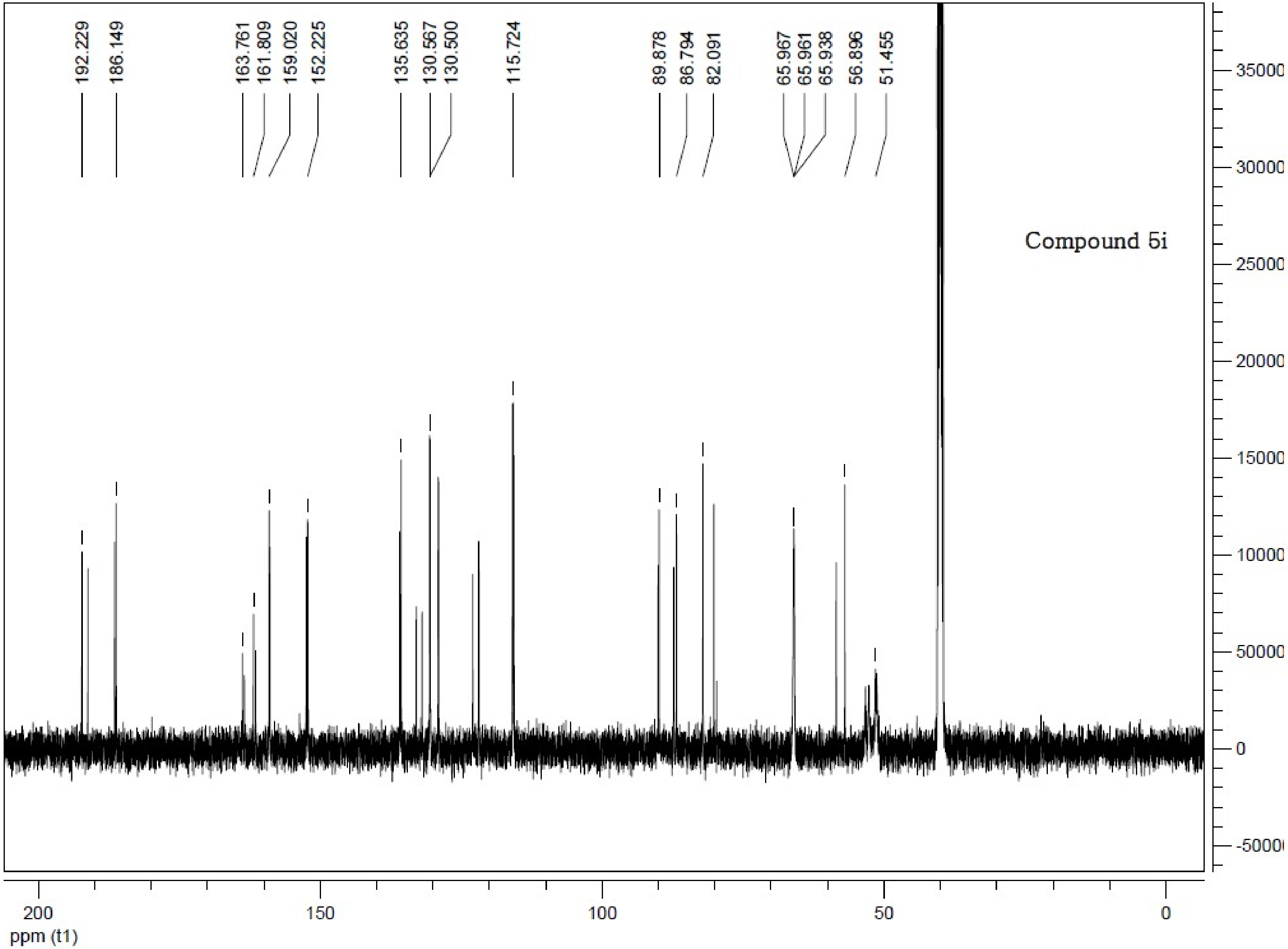

192.229
186.149
163.761
161.809
159.020
152.225
135.635
130.567
130.500
115.724
89.878
86.794
82.091
65.967
65.961
65.938
56.896
51.455
Compound 5i
35000
30000
25000
20000
15000
10000
50000
0
-5000
200
150
100
50
0
ppm (t1)

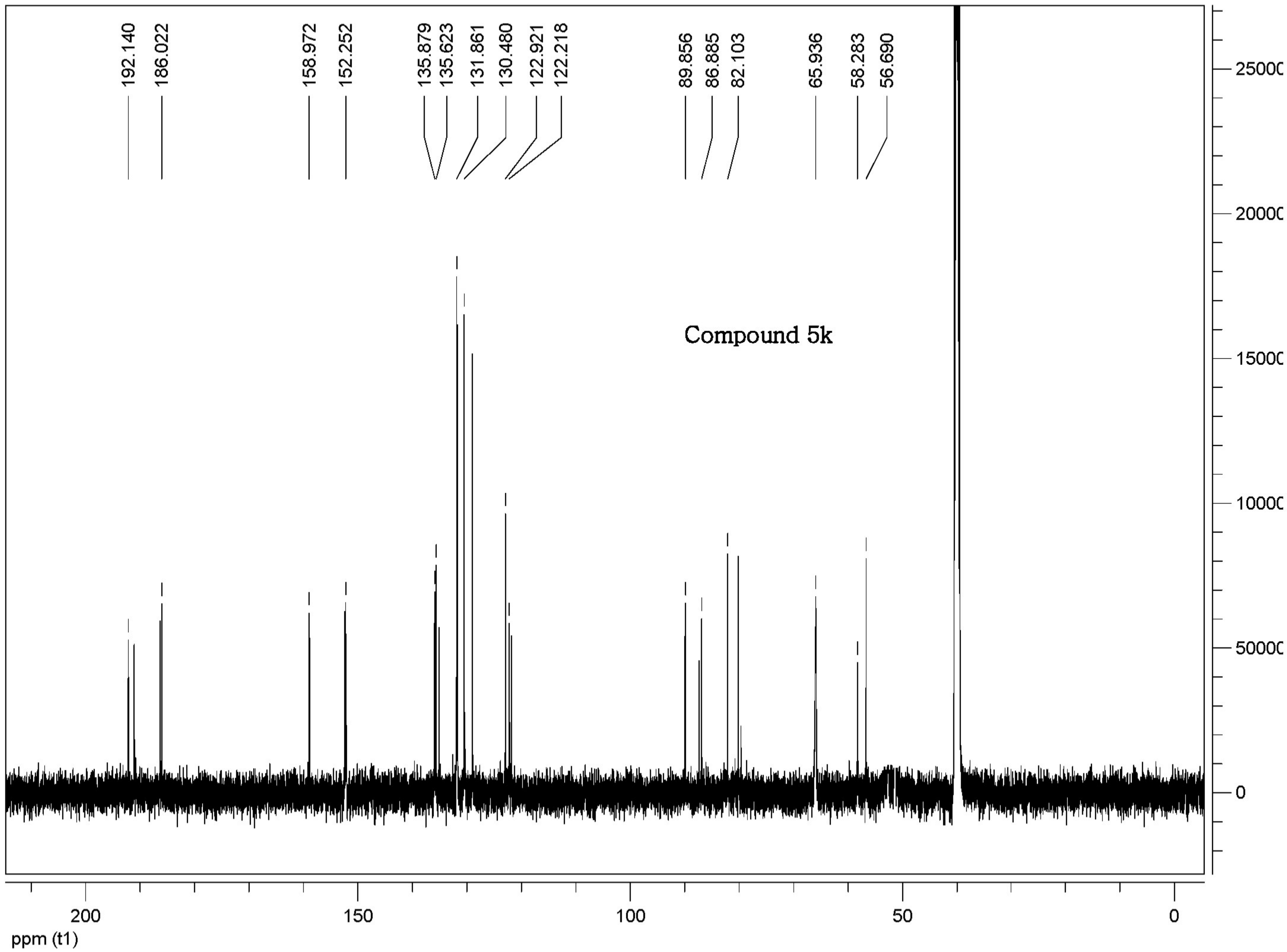
192.140
186.022
158.972
152.252
135.879
135.623
131.861
130.480
122.921
122.218
89.856
86.885
82.103
65.936
58.283
56.690
Compound 5k
25000
20000
15000
10000
50000
0
200
150
100
50
0
ppm (t1)

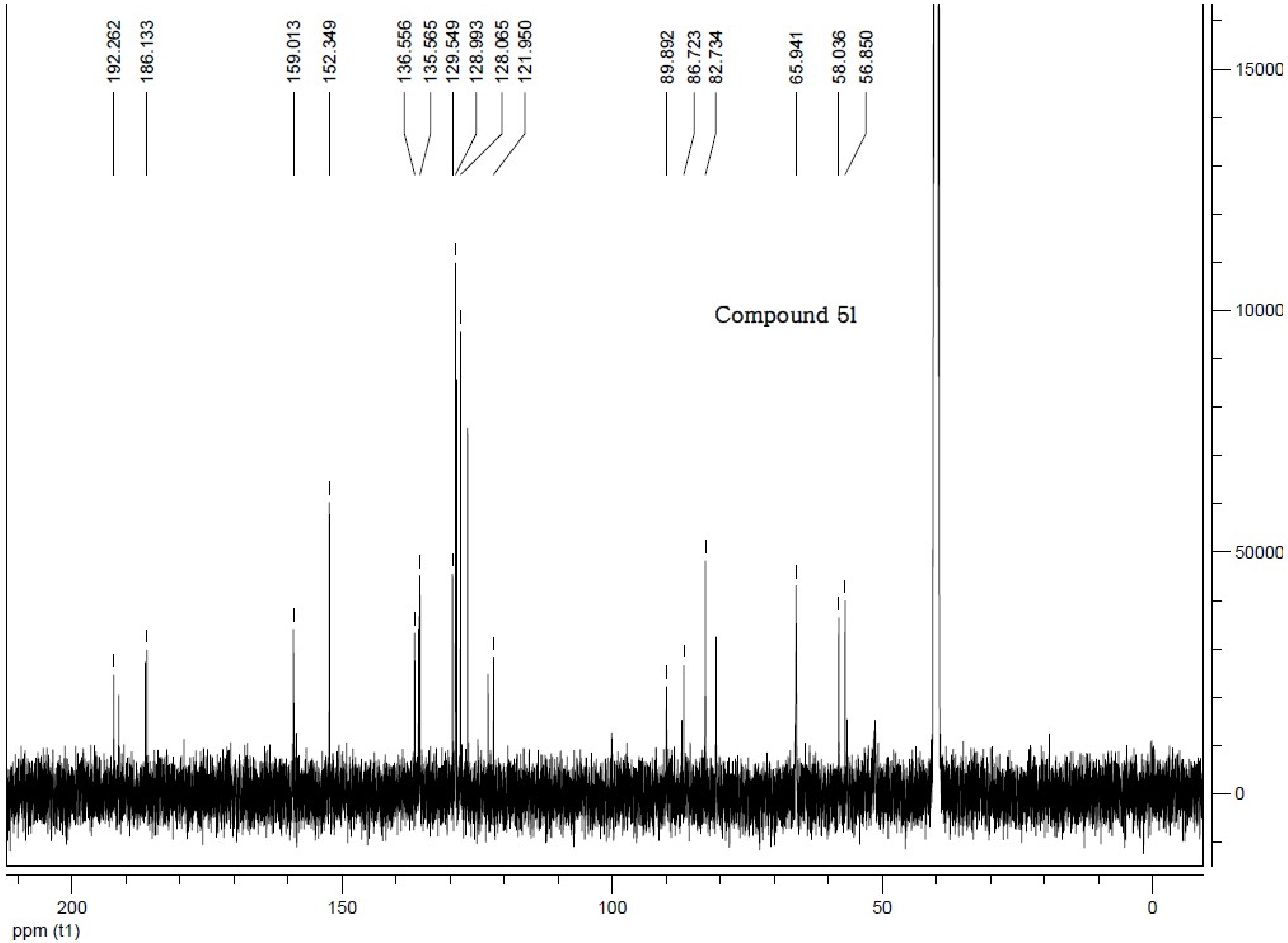

192.262
186.133
159.013
152.349
136.556
135.565
129.549
128.993
128.065
121.950
89.892
86.723
82.734
65.941
58.036
56.850
Compound 51
15000
10000
50000
0
200
150
100
50
0
ppm (t1)

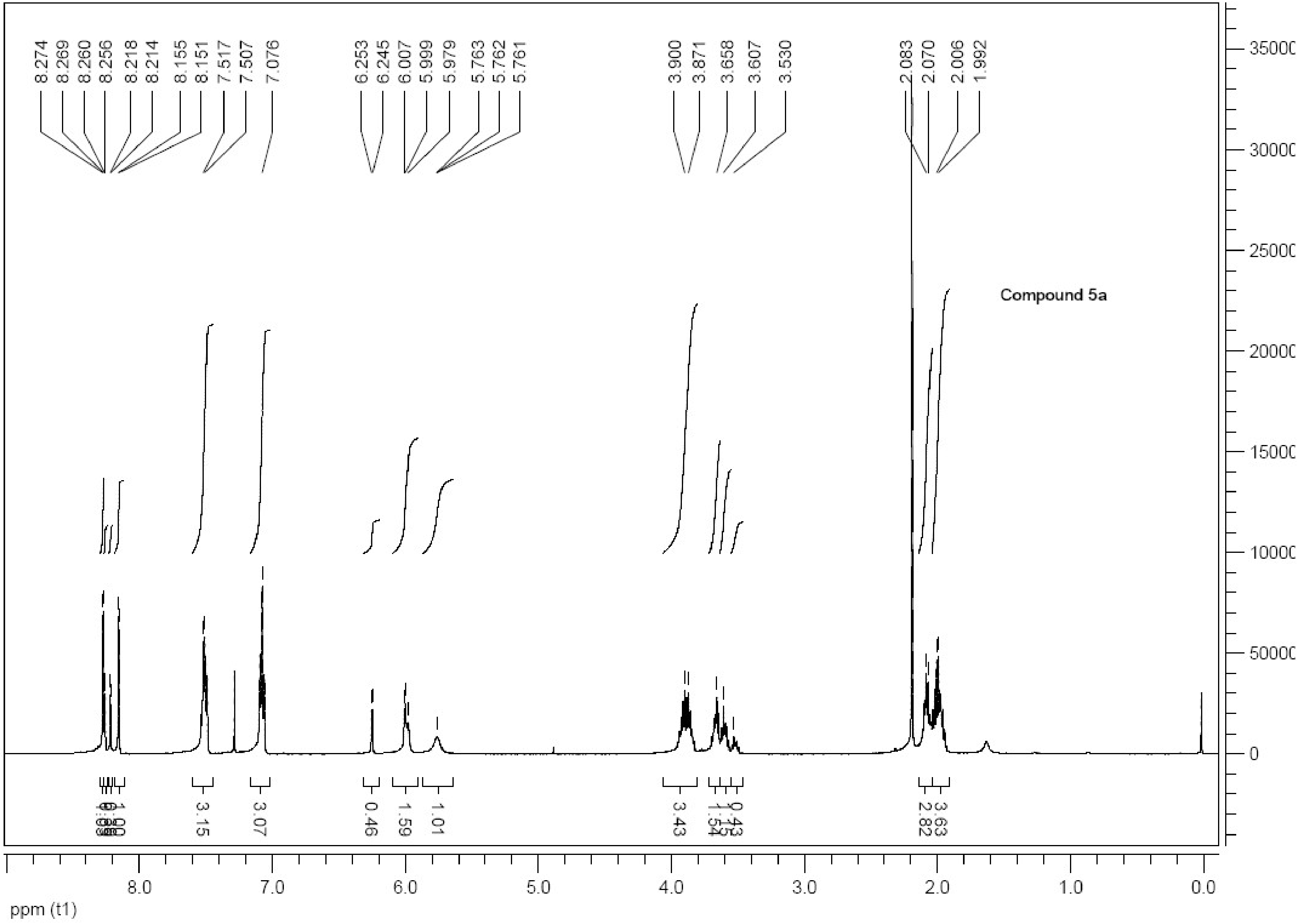
8.274
8.269
8.260
8.256
8.218
8.214
8.155
8.151
7.517
7.507
7.076
6.253
6.245
6.007
5.999
5.979
5.763
5.762
5.761
3.900
3.871
3.658
3.607
3.530
2.083
2.070
2.006
1.992
Compound 5a
3.15
3.07
0.46
1.59
1.01
3.43
2.82
3.63
8.0
7.0
6.0
5.0
4.0
3.0
2.0
1.0
0.0
ppm (t1)

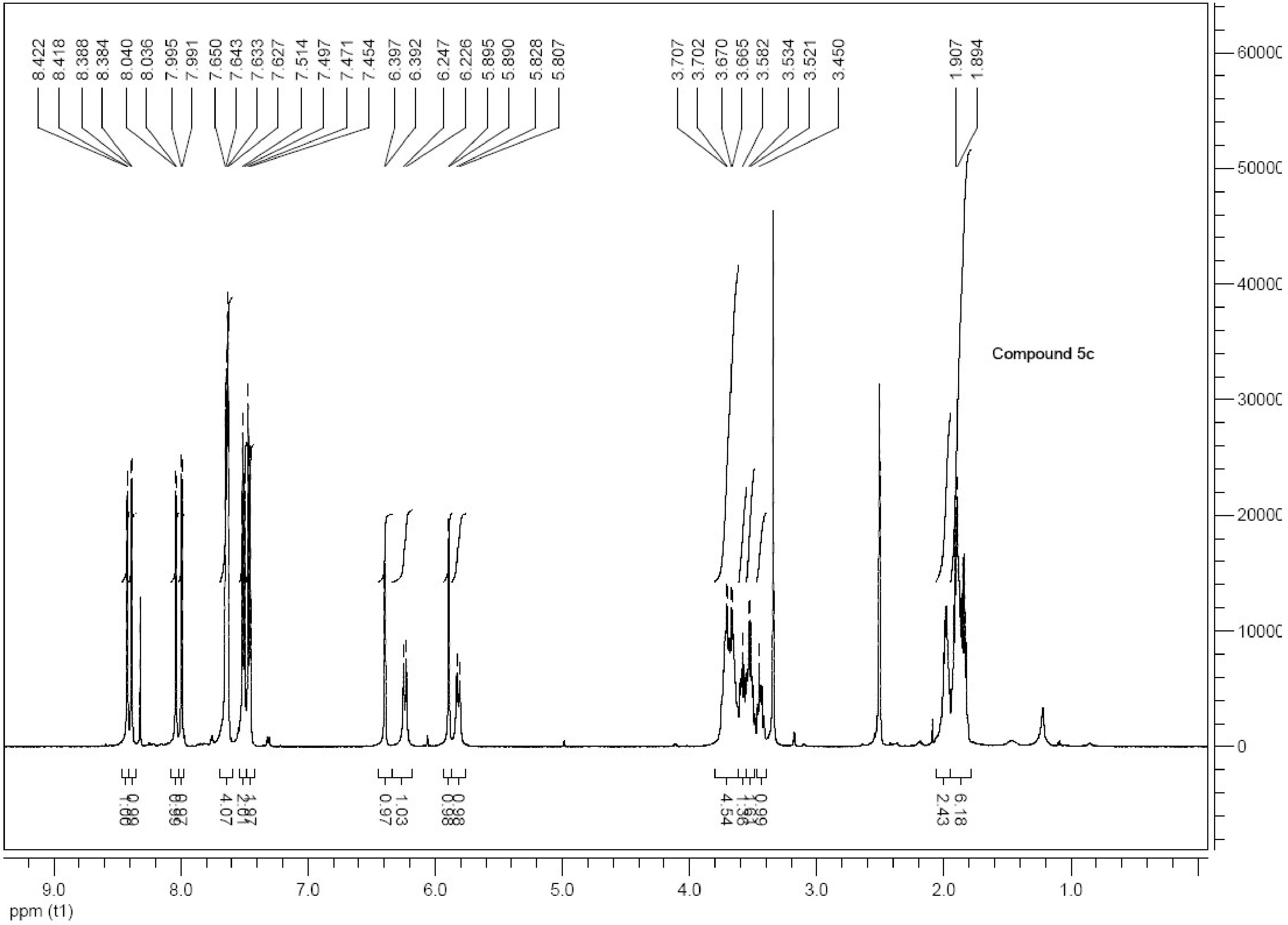
8.422
8.418
8.388
8.384
8.040
8.036
7.995
7.991
7.650
7.643
7.633
7.627
7.514
7.497
7.471
7.454
6.397
6.392
6.247
6.226
5.895
5.890
5.828
5.807
3.707
3.702
3.670
3.665
3.582
3.534
3.521
3.450
1.907
1.894
Compound 5c
4.07
0.97
1.03
0.98
4.54
0.99
2.43
6.18
60000
50000
40000
30000
20000
10000
0
9.0
8.0
7.0
6.0
5.0
4.0
3.0
2.0
1.0
ppm (t1)

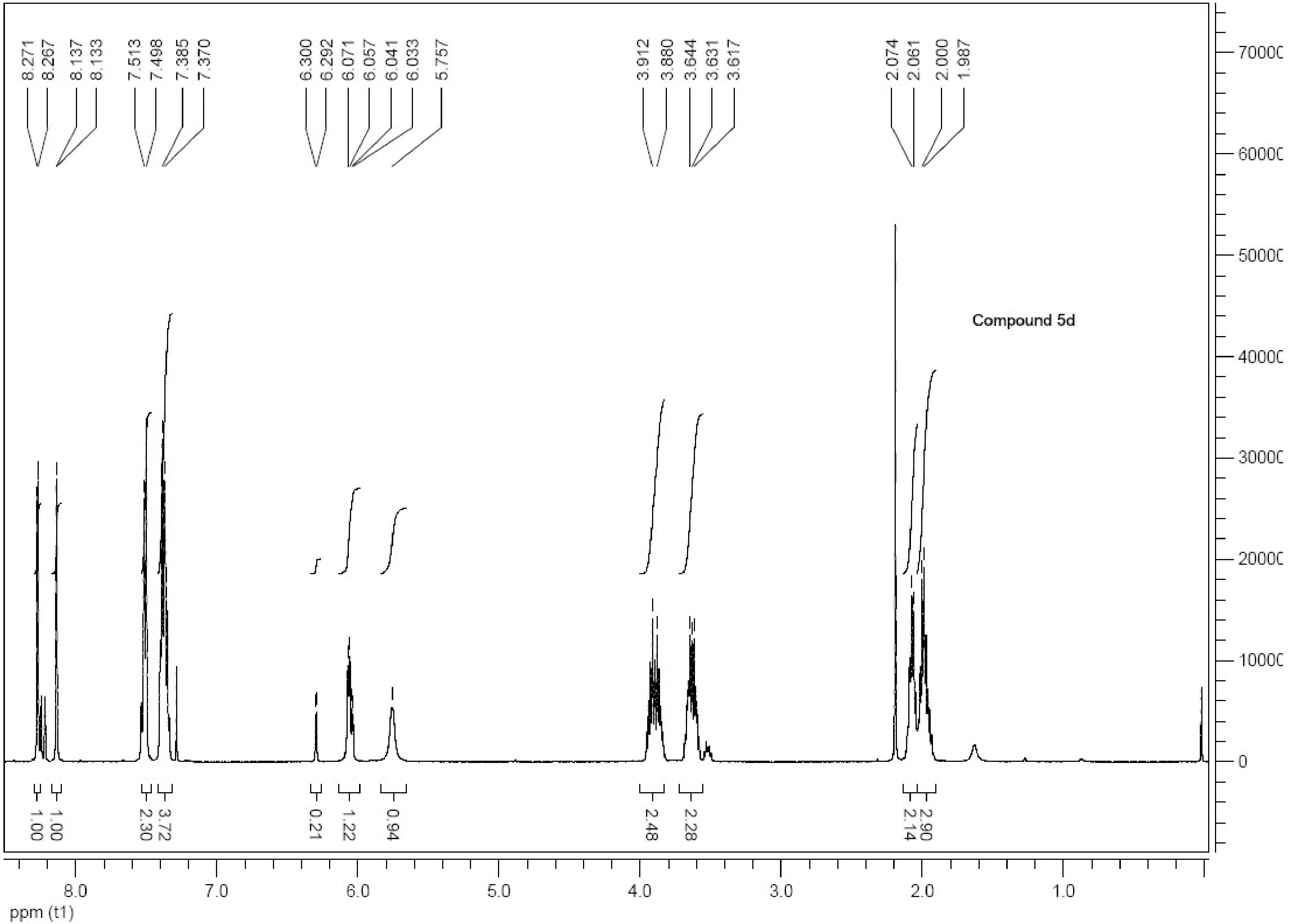
8.271
8.267
8.137
8.133
7.513
7.498
7.385
7.370
6.300
6.292
6.071
6.057
6.041
6.033
5.757
3.912
3.880
3.644
3.631
3.617
2.074
2.061
2.000
1.987
Compound 5d
1.00
1.00
2.30
3.72
0.21
1.22
0.94
2.48
2.28
2.14
2.90
ppm (t1)

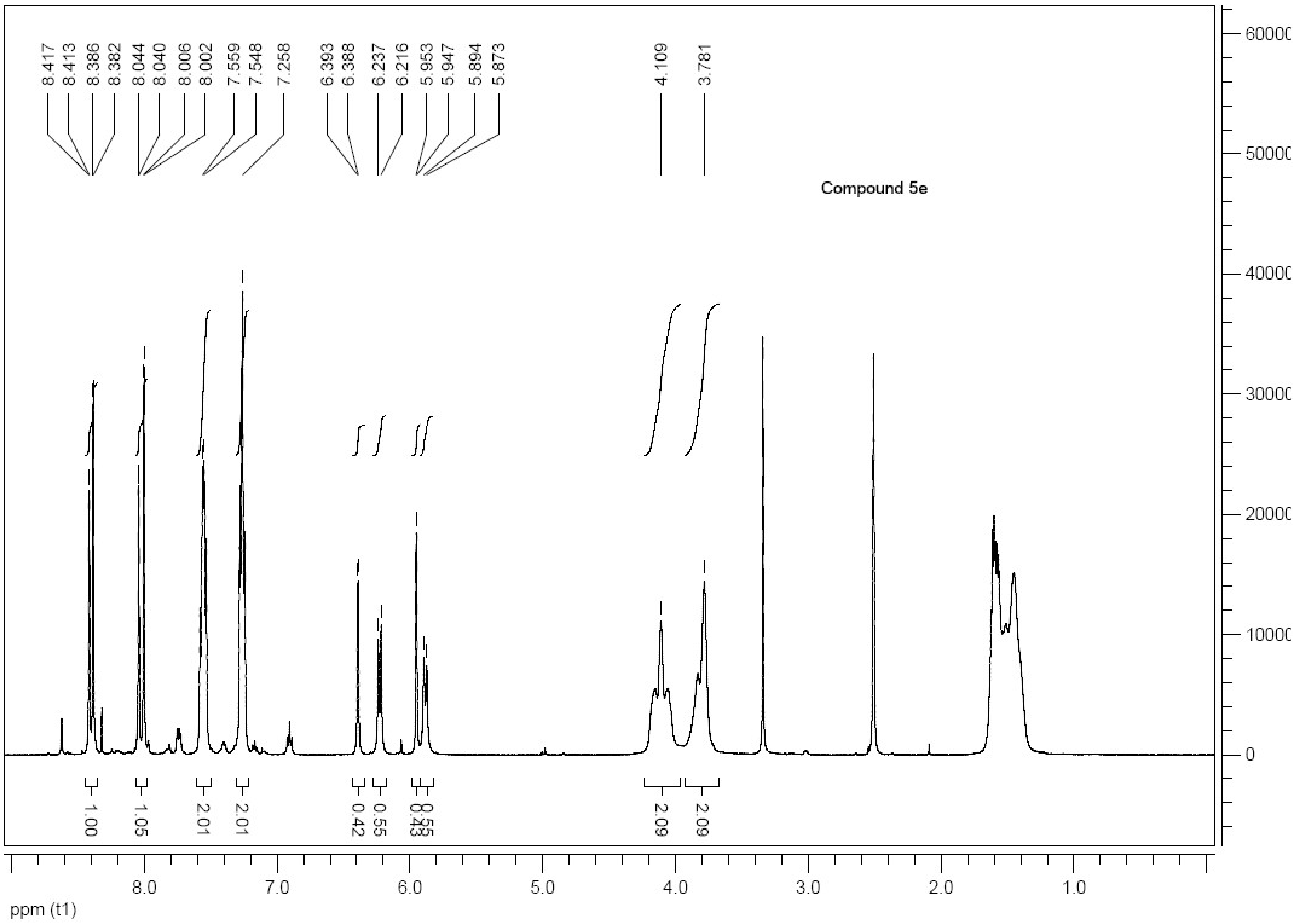
8.417
8.413
8.386
8.382
8.044
8.040
8.006
8.002
7.559
7.548
7.258
6.393
6.388
6.237
6.216
5.953
5.947
5.894
5.873
4.109
3.781
Compound 5e
1.00
1.05
2.01
2.01
0.42
0.55
0.43
0.55
2.09
2.09
ppm (t1)

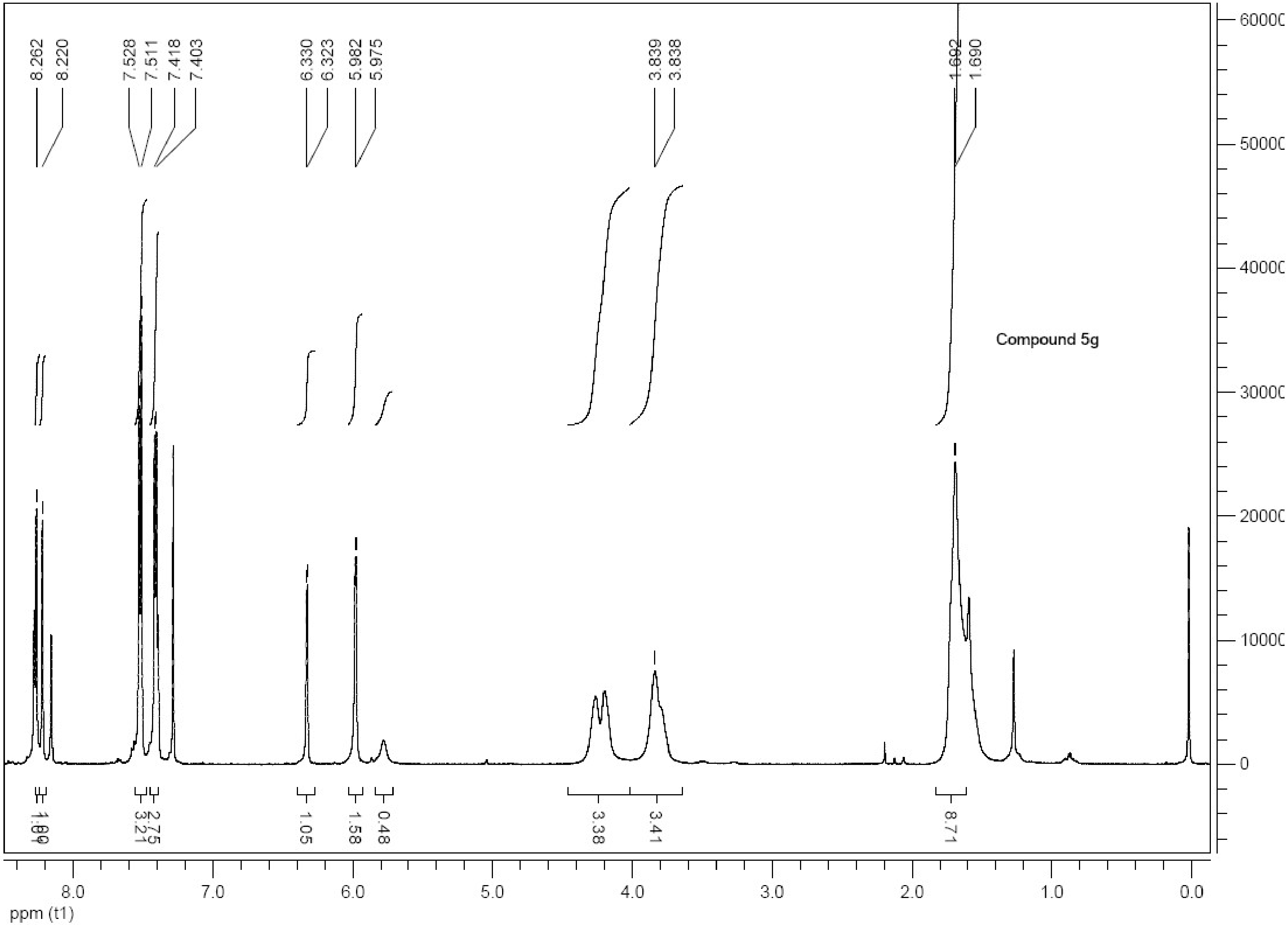
8.262
8.220
7.528
7.511
7.418
7.403
6.330
6.323
5.982
5.975
3.839
3.838
1.692
1.690
Compound 5g
3.21
2.75
1.05
1.58
0.48
3.38
3.41
8.71
8.0
7.0
6.0
5.0
4.0
3.0
2.0
1.0
0.0
ppm (t1)

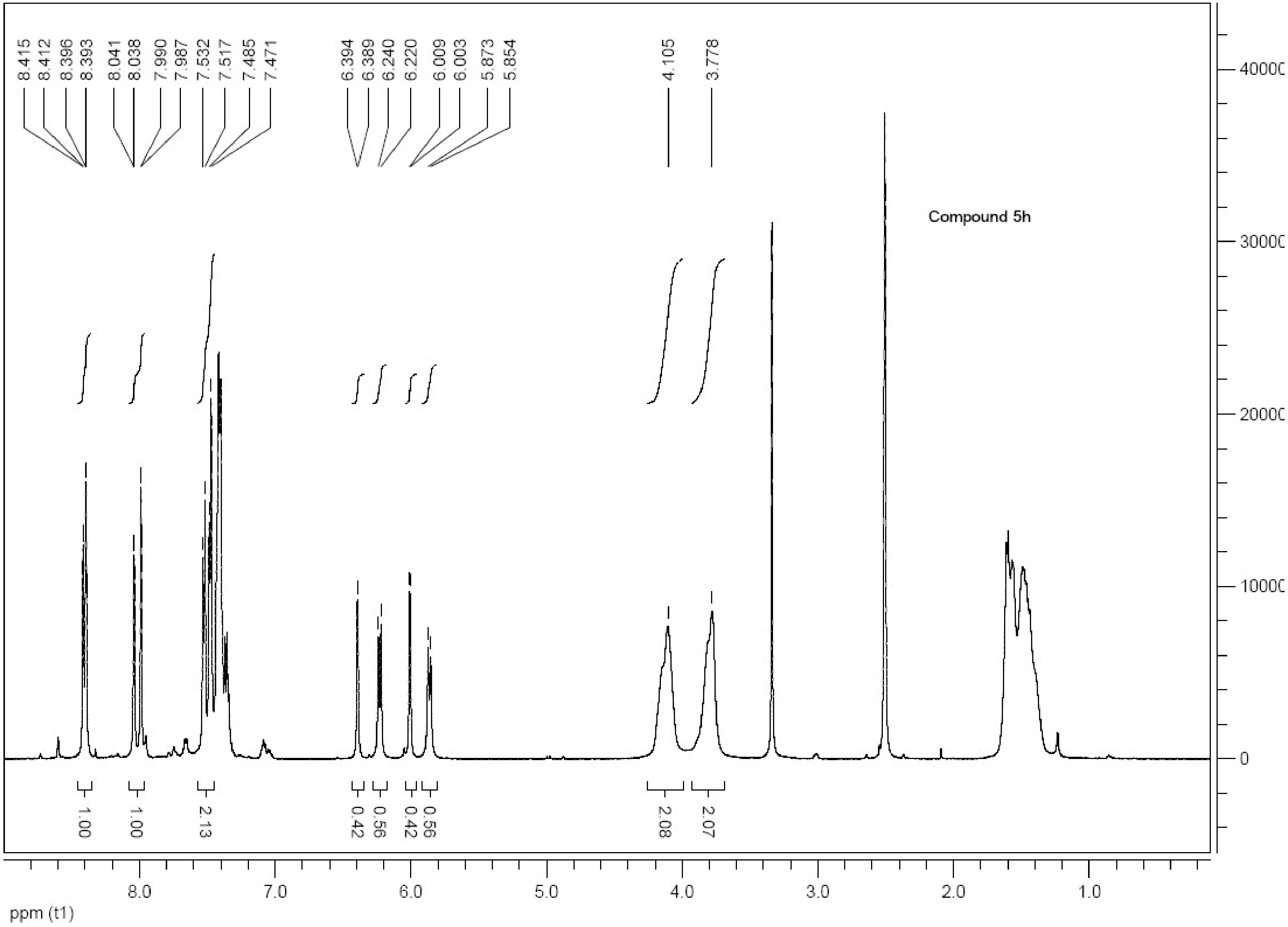

8.415
8.412
8.396
8.393
8.041
8.038
7.990
7.987
7.532
7.517
7.485
7.471
6.394
6.389
6.240
6.220
6.009
6.003
5.873
5.854
4.105
3.778
Compound 5h
1.00
1.00
2.13
0.42
0.56
0.42
0.56
2.08
2.07
8.0
7.0
6.0
5.0
4.0
3.0
2.0
1.0
ppm (t1)
40000
30000
20000
10000
0

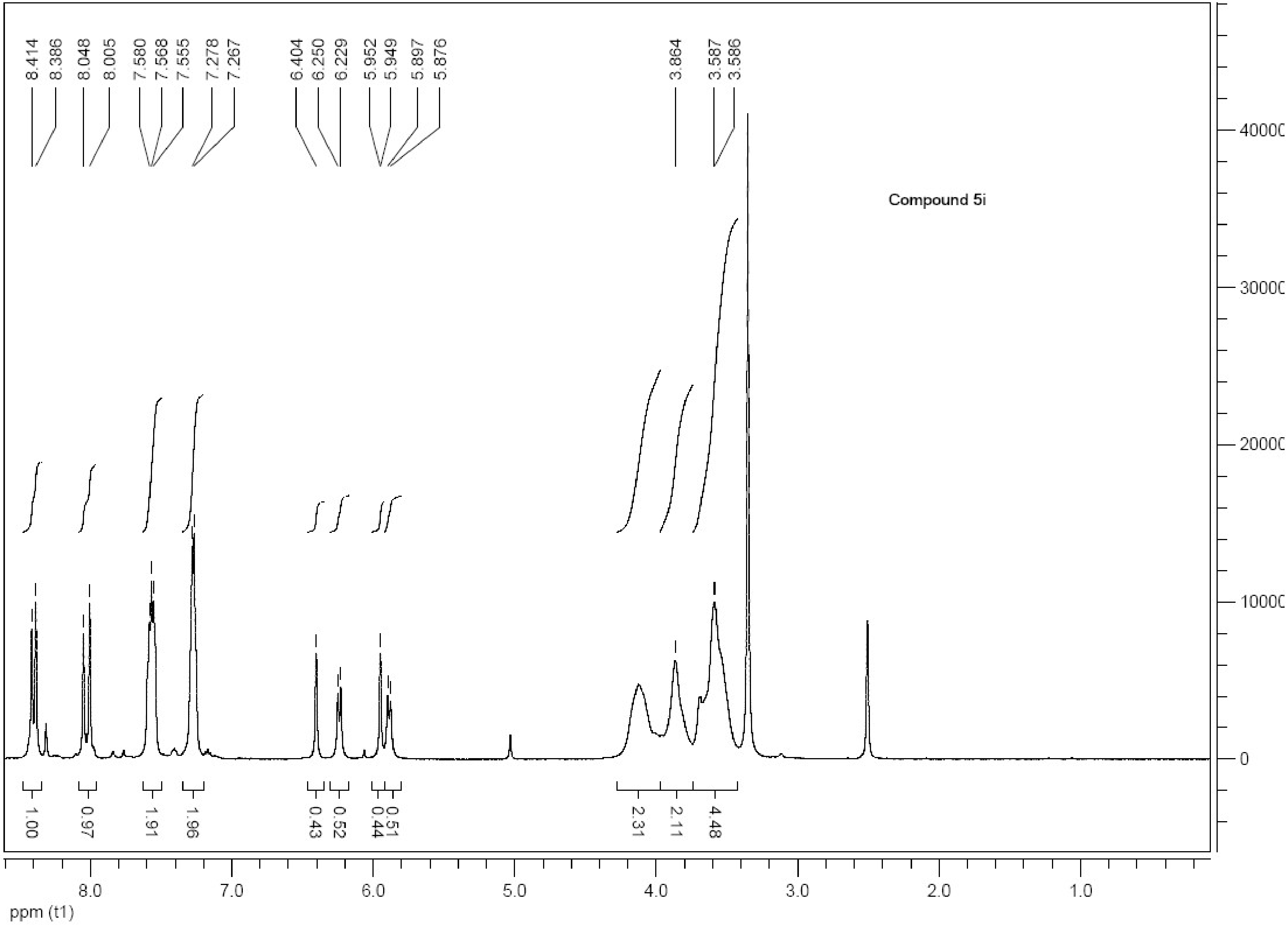
8.414
8.386
8.048
8.005
7.580
7.568
7.555
7.278
7.267
6.404
6.250
6.229
5.952
5.949
5.897
5.876
3.864
3.587
3.586
Compound 5i
40000
30000
20000
10000
0
1.00
0.97
1.91
1.96
0.43
0.52
0.44
0.51
2.31
2.11
4.48
8.0
7.0
6.0
5.0
4.0
3.0
2.0
1.0
ppm (t1)

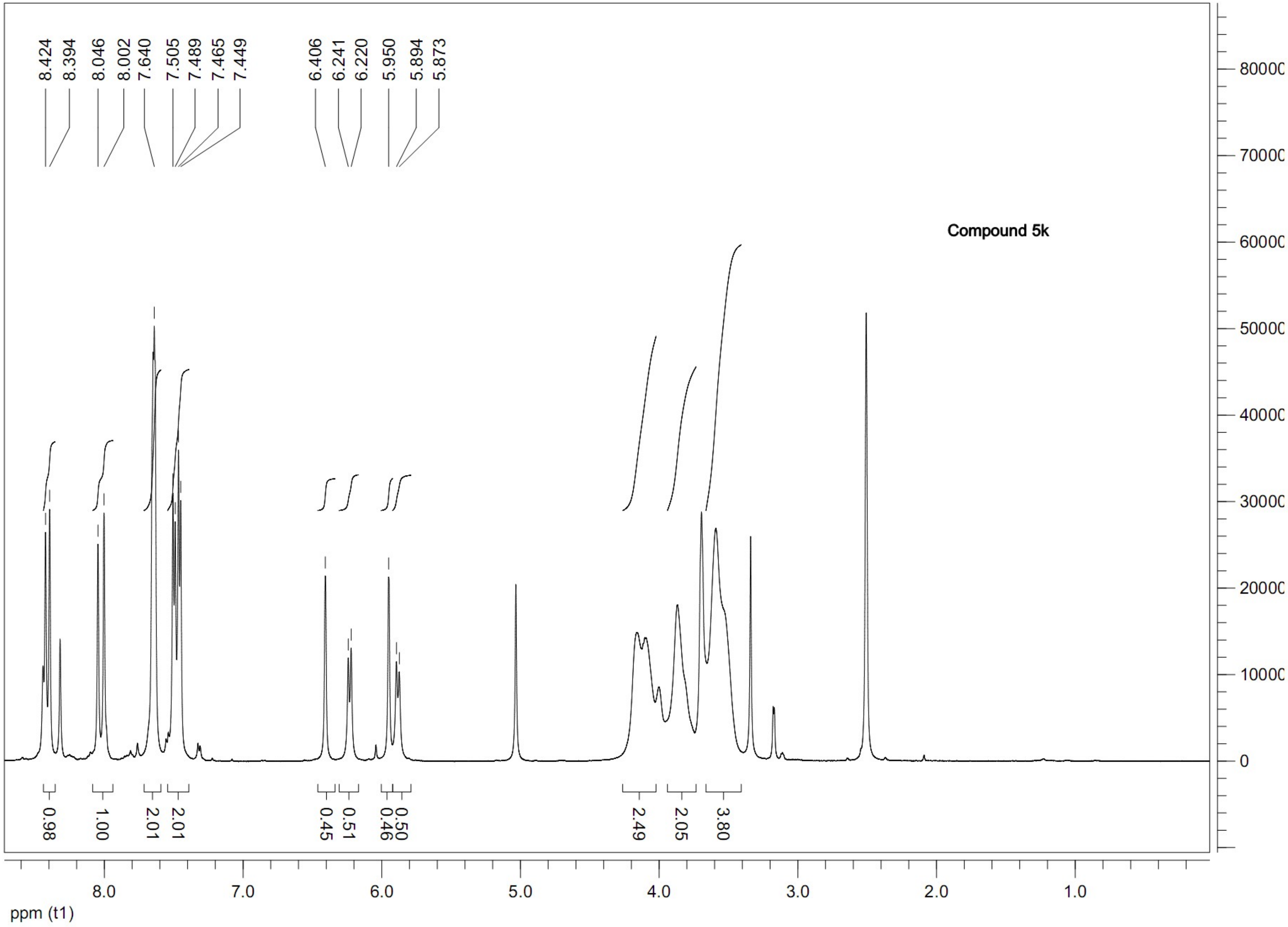
8.424
8.394
8.046
8.002
7.640
7.505
7.489
7.465
7.449
6.406
6.241
6.220
5.950
5.894
5.873
Compound 5k
0.98
1.00
2.01
2.01
0.45
0.51
0.46
0.50
2.49
2.05
3.80
8.0
7.0
6.0
5.0
4.0
3.0
2.0
1.0
ppm (t1)

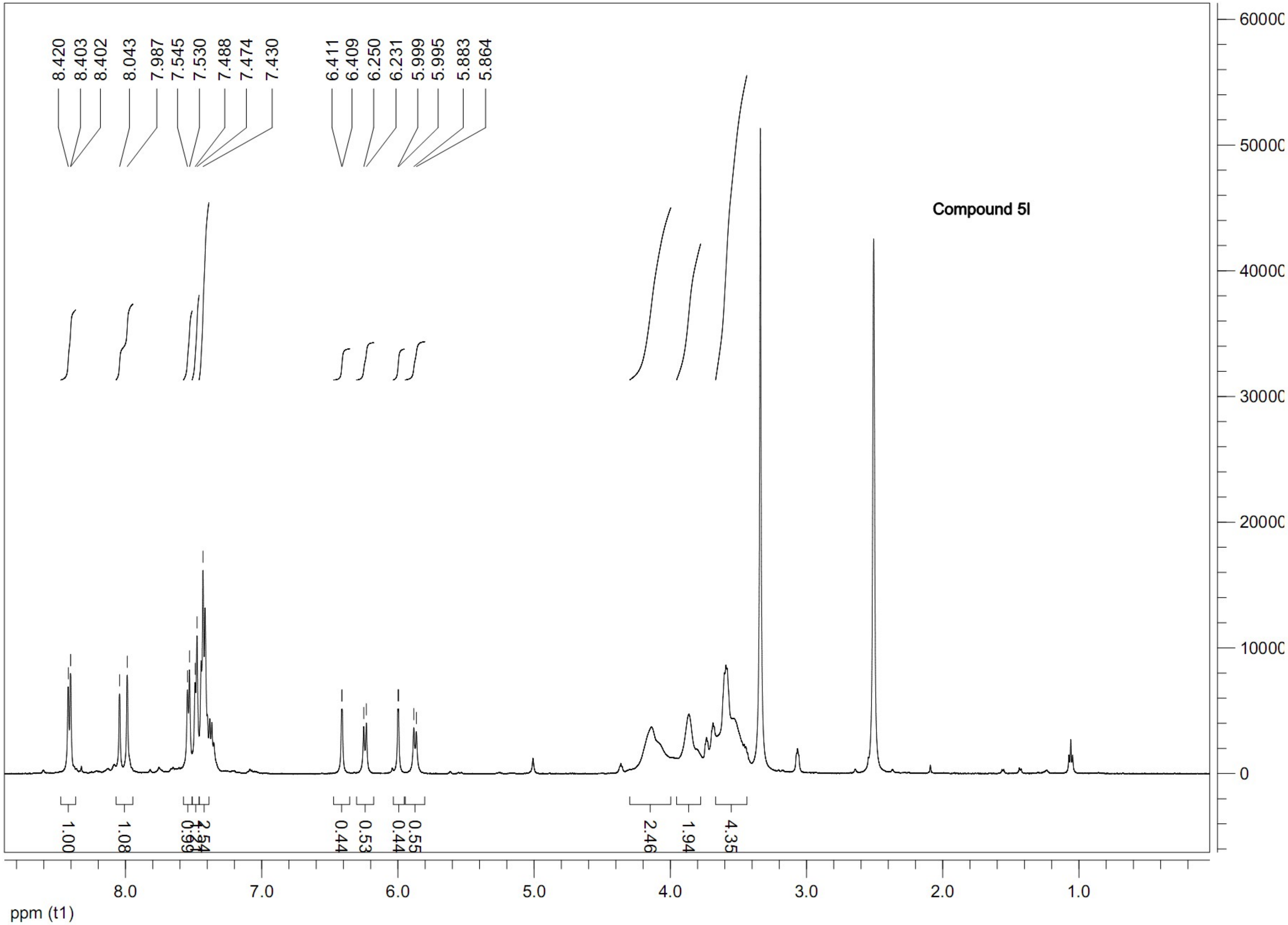
8.420
8.403
8.402
8.043
7.987
7.545
7.530
7.488
7.474
7.430
6.411
6.409
6.250
6.231
5.999
5.995
5.883
5.864
Compound 5l
1.00
1.08
2.46
1.94
4.35
0.44
0.53
0.44
0.55
8.0
7.0
6.0
5.0
4.0
3.0
2.0
1.0
ppm (t1)
